# *De Novo* Assembly of a Transcriptome for *Calanus finmarchicus* (Crustacea, Copepoda) – The Dominant Zooplankter of the North Atlantic Ocean

**DOI:** 10.1371/journal.pone.0088589

**Published:** 2014-02-19

**Authors:** Petra H. Lenz, Vittoria Roncalli, R. Patrick Hassett, Le-Shin Wu, Matthew C. Cieslak, Daniel K. Hartline, Andrew E. Christie

**Affiliations:** 1 Békésy Laboratory of Neurobiology, Pacific Biosciences Research Center, University of Hawaii at Manoa, Honolulu, Hawaii, United States of America; 2 Department of Biological Sciences, Ohio University, Athens, Ohio, United States of America; 3 National Center for Genome Analysis Support, UITS, Indiana University, Bloomington, Indiana, United States of America; Stazione Zoologica, Italy

## Abstract

Assessing the impact of global warming on the food web of the North Atlantic will require difficult-to-obtain physiological data on a key copepod crustacean, *Calanus finmarchicus*. The *de novo* transcriptome presented here represents a new resource for acquiring such data. It was produced from multiplexed gene libraries using RNA collected from six developmental stages: embryo, early nauplius (NI-II), late nauplius (NV-VI), early copepodite (CI-II), late copepodite (CV) and adult (CVI) female. Over 400,000,000 paired-end reads (100 base-pairs long) were sequenced on an Illumina instrument, and assembled into 206,041 contigs using Trinity software. Coverage was estimated to be at least 65%. A reference transcriptome comprising 96,090 unique components (“comps”) was annotated using Blast2GO. 40% of the comps had significant blast hits. 11% of the comps were successfully annotated with gene ontology (GO) terms. Expression of many comps was found to be near zero in one or more developmental stages suggesting that 35 to 48% of the transcriptome is “silent” at any given life stage. Transcripts involved in lipid biosynthesis pathways, critical for the *C. finmarchicus* life cycle, were identified and their expression pattern during development was examined. Relative expression of three transcripts suggests wax ester biosynthesis in late copepodites, but triacylglyceride biosynthesis in adult females. Two of these transcripts may be involved in the preparatory phase of diapause. A key environmental challenge for *C. finmarchicus* is the seasonal exposure to the dinoflagellate *Alexandrium fundyense* with high concentrations of saxitoxins, neurotoxins that block voltage-gated sodium channels. Multiple contigs encoding putative voltage-gated sodium channels were identified. They appeared to be the result of both alternate splicing and gene duplication. This is the first report of multiple Na_V_1 genes in a protostome. These data provide new insights into the transcriptome and physiology of this environmentally important zooplankter.

## Introduction

The recent crash of the cod fisheries in the North Sea has thrown a spotlight on a key prey of larval cod: the calanoid copepod *Calanus finmarchicus* [1]. The population of *C. finmarchicus* has been severely impacted in this part of its range, presumably because current environmental conditions are preventing it from completing its life cycle [2]. Individual-based population modeling has been an important approach to discover how physical, chemical and biological factors influence *C. finmarchicus* population dynamics [3]. These models have been hampered by a lack of adequate physiological information, leading to repeated calls for a better understanding of the physiological ecology of this planktonic organism [3]–[6]. However, because this species inhabits a 3-dimensional space that spans thousands of kilometers horizontally and a kilometer in depth across the open ocean, the resultant inaccessibility limits studies on the physiological ecology across habitats, seasons, climate (decadal oscillations, global climate change) and other environmental factors. A complex life history (including facultative diapause) and small size, as well as handling stress and time delays associated with collections, further limit *C. finmarchicus* and other key plankton as subjects for physiological studies. Thus, alternative approaches need to be developed for assessing physiological state in this species [7]. The application of gene expression pattern analysis offers one promising solution. Timely gene expression profiles encompassing a broad range of physiological processes might be obtained by combining RNA-Seq technologies with *in situ* preservation techniques [8]. Recent studies using subtractive hybridization, microarray and quantitative real-time polymerase chain reaction (qPCR) have demonstrated the value of gene expression studies in identifying differences in physiological state for individuals collected from different depths or differing in morphotype [9]–[11]. Global gene expression can identify biological, cellular and molecular processes that are regulated developmentally, seasonally and/or environmentally, and may thus provide key data for individual-based models.

Next-generation sequencing (e.g. 454 and Illumina platforms) has opened opportunities for developing molecular resources for non-model species that are of biological and economic interest, but which lack reference genomes [12]. Crustaceans, including copepods, are among the important invertebrates for which genomic resources are still limited [7]; a single crustacean genome is currently available publicly, *e.g.* that for the highly-derived cladoceran, *Daphnia pulex* [13]. One barrier to crustacean sequencing projects has been that many of the potential target species, including *C. finmarchicus*, have large genomes (C-values >5 pg*;* www.genomesize.com). Of course, this also raises the question of how these large genomes and transcriptomes differ from the much smaller one of *D. pulex* (C-value <0.4 pg; www.genomesize.com). Next-generation sequencing and the development of software programs to assemble the resultant short sequence reads make it possible to obtain transcriptomes for organisms with large genomes, as the transcriptomes are, in general, much smaller in terms of their nucleotide content [12], and they are more closely linked to physiological state. Thus, deep sequencing using RNA-Seq technology followed by *de novo* assembly has become an alternative to genome sequencing in the development of resources for protein discovery, developmental and physiological studies, and phylogenetic and evolutionary analyses for these organisms. With assembly programs like Trinity [14], [15], this approach can be used to identify rare transcripts and splice variants, and to formulate new hypotheses with respect to isoforms originating from single or multiple genes at a level not practical with conventional approaches in non-model organisms.

In the present study, we used Illumina sequencing technology and *de novo* assembly to generate a transcriptome for *C. finmarchicus*. Six multiplexed gene libraries derived from RNA from individuals at different life stages were sequenced in a single lane in order to include genes differentially expressed over the course of development. Depth and quality of the assembled transcripts were determined using a combination of global and targeted annotation. Since the build-up of lipid stores is a critical component of the *C. finmarchicus* life cycle, we used the resultant *de novo* assembly for targeted gene discovery and expression analysis focused on transcripts involved in lipid biosynthesis pathways. One result of this approach was the identification of transcripts with developmental expression patterns that are consistent with their involvement in the preparatory phase of diapause. Another key environmental challenge for some *C. finmarchicus* populations is the seasonal exposure to a toxic dinoflagellate (*Alexandrium fundyense*) with high concentrations of saxitoxins, neurotoxins that block voltage-gated sodium channels [16]. Efforts to sequence the voltage-gated sodium channel using traditional PCR and cloning techniques have been unsuccessful in this species (M.C. Chapline and A.E. Christie, personal communication). We identified and characterized multiple sequences encoding putative voltage-gated sodium channels by mining the *de novo* transcriptome. These transcripts appeared to be the result of both alternative splicing and gene duplication in *C. finmarchicus*. In summary, the data presented in our study represent a powerful new resource for protein discovery and stage-specific gene expression analysis in *C. finmarchicus*, which will provide important insights needed to understand the physiological ecology of this ecologically critical North Atlantic zooplankter.

## Materials and Methods

### Sample Preparation and Sequencing

Development in *C. finmarchicus* consists of an embryonic stage that occurs within the egg followed by six naupliar (NI-NVI) and six copepodite (CI-CVI) stages. For the transcriptome described here, total RNA was obtained from six developmental samples of whole individuals (Table 1): embryo (egg), early nauplii (stages NI and NII), late nauplii (stages NV and NVI), early copepodites (stages CI and CII), pre-adults (stage CV) and adult females (stage CVI). Adult females and pre-adults (CV stage copepodites) were collected in June and July of 2011 from coastal waters near Mount Desert Rock, Gulf of Maine, NW Atlantic Ocean (Lat: 44° 2′N; Long: 68°3′W) as described previously [17]. Adult females (10 individuals) and sub-adults (6 lipid-rich stage CV individuals) were isolated from the 14-July-2011 field collection upon return to the laboratory, rinsed in filtered seawater, placed on a sieve to remove the seawater and transferred into RNA extraction buffer. Samples for the other developmental stages (embryos, nauplii and early copepodites) were obtained from laboratory-reared individuals. After collection, individual animals were transferred into 3.5 and 10 L containers of filtered natural seawater and held in an incubator maintained at 8–9°C and 12∶12 L:D. Cultures were fed three times per week on live *Rhodomonas baltica* and algal paste (Reed Mariculture Shellfish diet). Adult females and males were isolated from these holding containers and placed into brood chambers, fed on *R. baltica ad libitum*, and checked for eggs daily. Eggs were separated from the brood chambers and either prepared for RNA extraction (400 eggs), or transferred to small culturing jars (250 to 500 ml) and returned to the incubator. The jars were checked daily and nauplii and copepodites were staged. After nauplii reached the feeding stage (NIII) *R. baltica* was added. As cultures reached the target stages, individuals were harvested, transferred through several washes of filtered seawater, and then placed into RNA extraction buffer. The number of individuals in each sample was: 180 early nauplii, 50 late nauplii and 40 early copepodites.

**Table 1:** Summary of *Calanus finmarchicus* samples prepared for RNASeq showing developmental stages, numbers of individuals (# ind) used for RNA extraction, RNA extraction results (sample concentration in ng/µL), amount of total RNA used in library preparation (ng), and Illumina HiSeq sequencing yields in number of megabases (Mb) and number of 100 bp raw reads.

Sample	ID	# ind	RNA conc (ng/µL)	Library RNA (ng)	Sequencing Yields (Mb)	Raw Reads (#)
Embryo	7414	400	17	476	5,645	59,001,054
Early nauplius (NI-NII)	7412	185	5.6	157	6,690	70,036,535
Late nauplius (NV-NVI)	7413	50	14	392	6,762	71,064,356
Early copepodite (CI-CII)	7410	40	110	3,000	6,848	71,585,082
Late copepodite (CV)	7411	6	192	3,000	7,252	75,871,920
Adult female (CVI)	7415	10	630	3,000	6,501	67,910,746
Total						415,469,690

Total RNA was extracted using a QIAGEN RNeasy Plus Mini Kit (catalog # 74134) with Qiashredder (catalog # 79654) following the instructions of the manufacturer with a final elution volume of 30 µl. For sequencing, a single RNA sample was generated for each developmental stage (Table 1). Sample concentration and quality were checked using an Agilent model 2100 Bioanalyzer or an Agilent RNA 6000 Bioanalyzer Nano (Agilent Technologies). Total RNA samples were shipped on dry ice to the University of Georgia Genomics Facility (dna.uga.edu) for library preparation and sequencing. Double-stranded cDNA libraries were prepared from total RNA extracted using the TruSeq RNA sample preparation kit (Illumina catalog # RS-122-2001) following manufacturer’s instructions. Briefly, RNA samples were first purified with two oligo-dT selection (poly(A) enrichment using oligodT beds), and then fragmented and reverse transcribed into double-stranded complementary cDNA. The resulting six cDNA libraries were prepared with a 350 bp insert and primed using random hexamers. Each sample was tagged with an indexed adapter prior to shipping to Alpha Hudson Institute for Biotechnology (www.hudsonalpha.org) for sequencing. The samples were paired-end sequenced (100 bp) in a single lane using an Illumina HiSeq 2000 instrument.

### *De novo* Sequence Assembly and Mapping of Reads

Prior to assembly, raw reads were assessed for quality using FASTQC (v0.10.0) software. Over-represented sequences were checked using blastn and were found to be *C. finmarchicus* ribosomal RNA. These sequences, which were less than 10% of the raw read data, were removed. The random primer sequences (first 9 bases) were trimmed prior to assembly using FASTX Toolkit (version 0.013; http://hannonlab.cshl.edu/fastx_toolkit/) software. The resulting reads were then *de novo* assembled using Trinity 2012-03-17-IU_ZIH_TUNED software (http://trinityrnaseq.sourceforge.net/) [14], [15] on the National Center for Genome Analysis Support’s (NCGAS; Indiana University, Bloomington, IN, USA) Mason Linux cluster; each node of this computer system is composed of four Intel Xeon L7555 8-core processors running at 1.87 GHz with 512 GB of memory. For the assembly, reads from all six developmental stage samples were combined and the minimum sequence length in the assembly was set to 300 bp. Trinity comprises three separate software programs (“Inchworm”, “Chrysalis” and “Butterfly”), which process the data sequentially [14], [15]. The final output from Trinity is a large number of assembled FASTA sequences that are each identified by a unique Chrysalis component number (comp), followed by a “c” identifier, which is a Butterfly disconnected sub-graph designation, and a “seq” designation which is a Butterfly reconstructed sequence [15]. For simplicity, we refer to the individual assembled sequences as “contigs”, and the clustered components as “comps”. For assembly, the initial parameters of Trinity were set as follows: –seqType fq –bfly_opts “–edge-thr = 0.05” –kmer_method jellyfish –CPU 32–max_memory 20G –min_contig_length 300 - bflyHeapSpaceMax 8G –bflyGCThreads 4. The resulting *de novo* assembly was used to generate two transcriptomes, the “complete” assembly and the “reference” transcriptome. The complete assembly consisted of all contigs. The reference transcriptome included only unique comps; in situations in which a given numbered unique comp contained multiple contigs, the longest contig was selected for the reference. It should be noted that this procedure can result in the omission of occasional distinct genes grouped by the software under a single comp number. The reference transcriptome was annotated using Blast2GO (see below). Both transcriptomes were used to map reads from each of the stage-specific samples, as well as in a combined sample, using Bowtie (version, 2.0.6; default settings of two mismatches) software [18]. Before Bowtie mapping, reads were again quality filtered using FASTX Toolkit software, with a Phred quality score of 20 used as a limit. Low quality reads (fewer than 8%) were removed from the dataset.

Additional assemblies using the same settings were performed on the individual stage-specific samples, as well as subsets of all reads starting at 6 million reads to assess assembly statistics as a function of developmental stage and sequencing depth. Subsets were acquired by successively extracting every other read from a fastq file using a custom written Perl script (fastqDivide.pl, available at http://github.com/LenzLab/RNA-seq-scripts). Different “sequencing samples” were generated by further dividing subsets and/or recombining mutually exclusive subsets.

### Assembly Validation and Annotation

To determine the extent of coverage of the Trinity assembly, and to assess its similarity to those of other species, functional annotation was undertaken using Blast2GO (version 2.6.4) [19] for the reference transcriptome. For this analysis, the blastx algorithm was used to search against the NCBI non-redundant (nr) and SwissProt protein databases, which were downloaded (February 2013) onto a local Beowulf Linux computer cluster; a maximum E-value for annotation of 10^−3^ was employed in both searches. Gene ontology (GO) annotations for biological and molecular processes and cellular component were assigned using Blast2GO, here with a maximum E-value of 10^−6^ required for annotation.

To verify the coverage of our annotation results, we compared the proportion of sequences annotated in selected GO term groups to the proportion in these categories reported for the *Drosophila melanogaster* genome from a pre-computed GO annotation (http://www.b2gfar.org/showspecies?species=7227). Percentages of GO for biological process, molecular function and cellular component at ontology level 2 were calculated by dividing the total number of sequences annotated to a given GO term by the total number of annotated compounds (x100). A large percentage of genes were not expressed in any particular stage. Thus, for each developmental stage, we did a functional analysis of the “silent” transcripts. We determined the relative percentages of GO terms for biological process, molecular function and cellular component that were not expressed (≤2 mapped reads).

Targeted gene discovery was focused on two groups of transcripts encoding for: 1) putative proteins involved in lipid biosynthesis; and 2) voltage-gated sodium channels. This analysis was used to gain further insight into the completeness and quality of the assembly, as well as expression patterns during development and the biological significance of compounds with multiple sequences. In addition to searching the annotated reference transcriptome, the complete transcriptome assembly was downloaded to a TimeLogic DeCypher server at the Mount Desert Island Biological Laboratory (Salsbury Cove, Maine) and searched using the Tera-BLASTP algorithm for sequences that were putative homologs of a known protein query (typically ones from the fruit fly *D. melanogaster*). The C. *finmarchicus* nucleotide sequences identified in this manner were fully translated and then aligned with and checked manually for homology to the query protein. In addition, each deduced *Calanus* protein was used as the query in reciprocal BLAST analyses against 1) the annotated proteins in FlyBase and 2) the non-redundant proteins curated at NCBI to identify the most similar protein in each database as a second measure of annotation. Conserved regions were identified by aligning *C. finmarchicus* predicted proteins with the *D. melanogaster* sequence showing functional domains to confirm that each predicted protein possessed the correct structural hallmarks. Finally, in an attempt to assess the correctness of assembled nucleotide sequences, each was used as a query in a blastn search of the extant *C. finmarchicus* ESTs (<12,000 in total) [10] curated at NCBI for transcripts encoding identical or highly similar sequences. This targeted transcript discovery workflow was modified from one described in detail in several recent publications [17], [20], [21].

Sequence data and the de novo assembly have been submitted to the National Center of Biotechnology Information (NCBI; www.ncbi.nlm.nih.gov) under bioproject PRJNA236528.

## Results and Discussion

### Sequencing Results and Assembly

Illumina sequencing of the six *C. finmarchicus* developmental stage libraries (embryos, early nauplii, late nauplii, early copepodites, pre-adults and adult females) yielded over 400 million paired-end 100 bp reads, with an average of 69 million reads per developmental sample (Table 1). This species has a C-value (amount of DNA contained within a haploid nucleus) of 6.48 pg [22], which translates into an estimated genome size of more than 6,000 Mb (conversion factor 1 pg = 978 Mb) [23]. Assuming that only 7 to 10% of the *Calanus* genome is transcribed, the Illumina reads represent a sequencing coverage of approximately 60 to 90-fold for the combined samples. The number of base pairs used in the assembly exceeded 30 billion (number of reads multiplied by 91 bp [the 100 bp read trimmed of the 9 bp random primer sequence]), which generated a *de novo* assembly with a total length of 205 million base pairs (Table 2). Thus, the ratio of the number of base pairs in the assembled transcriptome to the total number of base pairs was approximately 150. These estimates suggest that the coverage obtained for the *C. finmarchicus* transcriptome is as deep or deeper than those obtained in other crustacean *de novo* transcriptomics studies [12], [24]–[26].

**Table 2:** Summary statistics for the *de novo* assembly of the *Calanus finmarchicus* transcriptome.

*C. finmarchicus* transcriptome assembly statistics	
Total number of trimmed and high quality raw reads assembled (91 bp) (91 bp)	401,836,653
Total number of assembled contigs	206,041
Minimum contig length (bp)	301
Average contig length (bp)	997
Maximum contig length (bp)	23,068
Total length of all contigs in assembly	205,480,825
Total GC count (bp)	88,329,861
GC Content for the whole assembly (%)	43
N50 (bp)	1,418
N25 (bp)	2,748
N75 (bp)	701

Raw reads (Table 1) were trimmed (9 bp) and over-represented and low quality reads were removed prior to *de novo* assembly using Trinity software.

**Figure 1:**
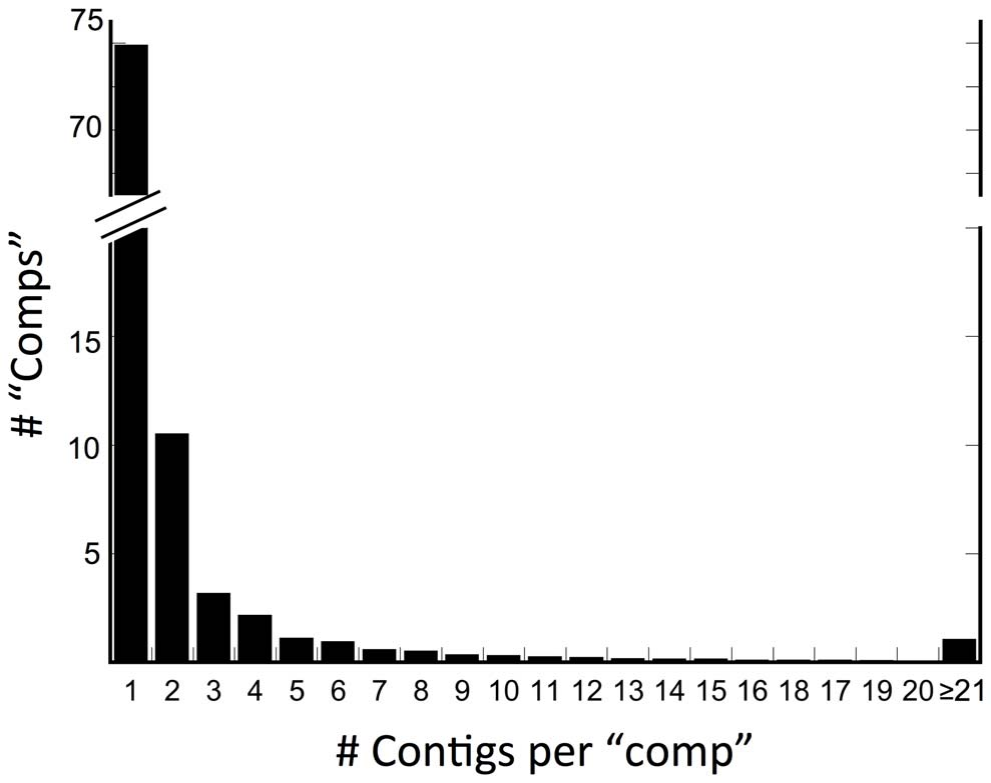
Frequency distribution of the number of contigs per unique component (“comp”). The *de novo* assembly generated 206,041 contigs that were organized into 96,090 unique comps. Number of contigs per comp ranged from 1 to over 1,500.

**Figure 2:**
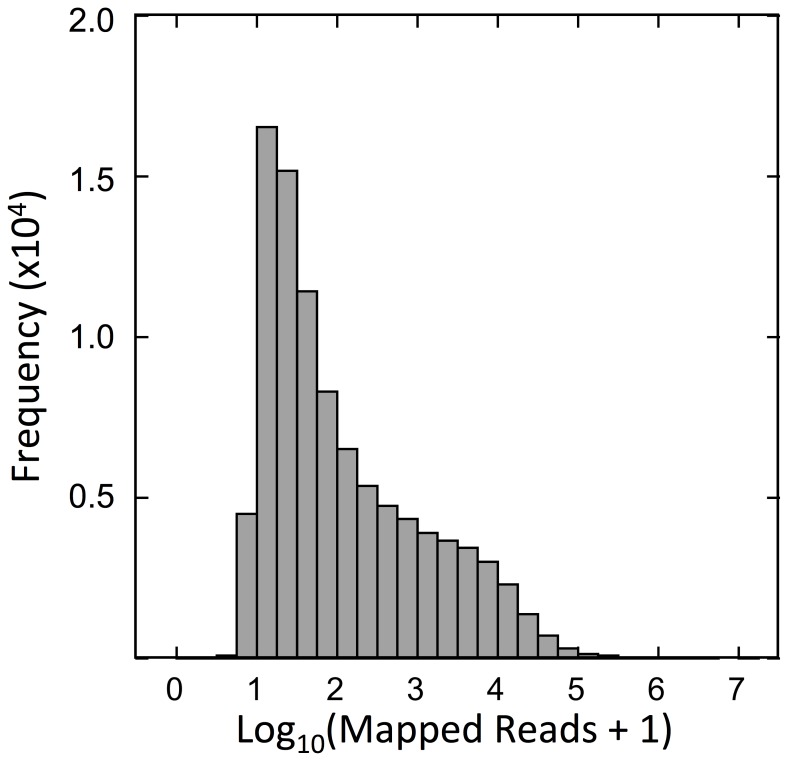
Frequency distribution of the number of mapped reads per reference transcript for all samples combined on a log scale. Trimmed and quality-filtered reads were mapped against the reference transcriptome comprising 96,090 comps.

Assembly of the Illumina reads by Trinity generated 206,041 contigs with an average length of 997 bp (Table 2). Half of these (N50) were at least 1,418 bp long and the longest contig was 23,068 bp long (Table 2). It contained 96,090 unique comps, of which 73,925 (77%) consisted of single contigs. The remaining comps consisted of multiple contigs and ranged from 2 to over 1,500 sequences (Figure 1). Mapping of the Illumina-generated reads against the complete, 206,041-sequence assembly yielded an overall alignment of 89% (Table 3; the missing reads presumably belonging to sequences below the 300 bp cut-off). However, given the redundancy found in the multiple contigs represented within some comps, a large percentage (44%) of reads mapped more than once (Table 3). Thus, the longest contig for each comp with multiple sequences, plus all singletons, were selected to produce a reference transcriptome of unique comps (96,090 sequences). When this sub-set was used as reference in the mapping step, the alignment rate decreased to 75% and the number of reads mapped >1 time decreased to 0.7%; Table 3). An analysis of the frequency distribution of number of reads showed that 75% of the predicted transcripts had 10 to 1000 reads mapped to them (Figure 2). Very few of the reference sequences had fewer than five (log_10_[reads+1] ≤0.75) or more than 10^5^ reads mapped to them (Figure 2).

**Table 3:** Summary of mapping results of *Calanus finmarchicus* RNASeq reads to whole assembly (206,042 contigs) and to the reference transcriptome (96,090 comps) using Bowtie software.

	Against whole assembly (206,041 contigs)	Against reference transcriptome (96,090 comps)
Reads for mapping	367,127,119	367,127,119
Total mapped reads	326,743,136	275,345,339
Overall alignment (%)	89	75
Reads mapped 1 time	147,034,411	206,509,004
Reads mapped 1 time (%)	45	75
Reads mapped >1 time	143,766,980	1,927,417
Reads mapped >1 time (%)	44	0.7

Reads used in the assembly (see Table 2) were filtered for quality using FASTX Toolkit, and low quality reads (8%) were removed prior to mapping.

**Figure 3:**
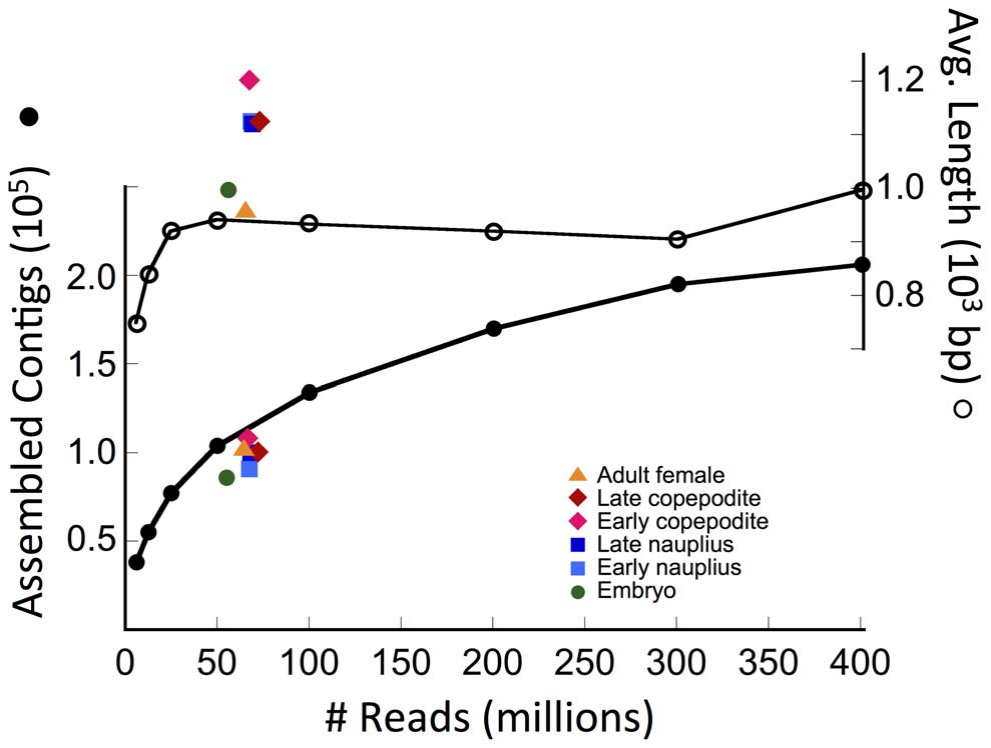
Number of assembled sequence contigs (black filled circle) and average lengths (open circle) of *de novo* assemblies generated by Trinity with increasing number of reads from all samples combined. Superimposed on the random read assemblies are the data for the assemblies generated from each of the six developmental stages (orange triangle: adult female [stage CVI]; red diamond: late copepodite [stage CV]; purple diamond: early copepodite [stages CI-CII]; dark blue square: late nauplius [stages NV-NVI]; light blue square: early nauplius [stages NI-NII]; green circle: embryo).

In order to obtain a measure of completeness of the assembly from the full set of reads, a series of *de novo* Trinity assemblies was generated using an increasing number of reads, from 6 million to the complete, 400,000,000+-reads dataset (Figure 3). The total number of contigs assembled increased steeply from 38,000 to 100,000 between 6 and 50 million reads (1.5 to 12.5% of total available reads; Figure 3). After this initial increase, the rate of increase declined (Figure 3). The number of unique comps in the assemblies also increased with number of reads (Table S1). In contrast, average sequence lengths were nearly constant, fluctuating between 900 and 1000 bp in the assemblies generated from 25 million reads and above (Figure 3; Table S1). The assembly statistics (average length, N25, N50, N75) obtained for the smaller data sets were similar over a similar range in number of reads (Table S1). These results suggest that good assemblies could be obtained from as few as 50 million reads, which is not surprising given that Trinity software is designed to generate good assemblies even when coverage is low [14]. However, the number of assembled contigs continued to increase with additional reads, suggesting that even at 400 million reads, rare transcripts were still missing from the *de novo* assembly. A 2-exponential fit to the data predicted an asymptote at ∼300,000 sequences, suggesting that the current assembly had ca. 65% of the total number of expected contigs. Independent estimates of completeness of the transcriptome were obtained through targeted protein discovery [17], [20], [21]. Searches for circadian proteins and the enzymes involved in amine biosynthesis identified putative transcripts for all expected proteins (100% coverage) [20], [21]. In contrast, searches for neuropeptide preprohormones and receptors yielded incomplete sets of predicted transcripts (52 to 60% of expected) [17]. Neuropeptide-encoding sequences are rare in whole organism transcriptomes since they are typically restricted to the nervous system and are expressed in a limited number of cells within this organ, including in *C. finmarchicus* [27]–[29].

*De novo* assemblies completed for the individual developmental stage samples are summarized in Table 4. The number of contigs obtained for each individual sample was lower than those generated by sub-samples of reads randomly selected from the combined samples (isolated points below curve in Figure 3, Table 4). The number of unique comps was also lower and ranged between 37,692 and 50,216 with 73 to 78% of these being singletons. This proportion of singletons was similar to the assembly of all reads combined. Average sequence lengths were longer than expected compared to the assembly statistics obtained for a similar number of randomly selected reads (isolated points above the curve in Figure 3). In addition, the longest contigs exceeded 20,000 bp in all stage-specific assemblies except for that derived from embryos (Table 4).

**Table 4:** Summary statistics for *de novo* assemblies generated for each sample separately.

	Embryo	Early nauplius NI-NII	Late nauplius NV-NVI	Early copepodite CI-CII	Late copepodite CV	Adult Female CVI
Assembled contigs (#)	86,385	91,413	100,496	108,759	100,841	103,455
Min. contig length (bp)	301	301	301	301	301	301
Avg. contig length (bp)	997	1,125	1,120	1,202	1,125	961
Max. contig length (bp)	14,977	24,548	25,122	26,420	24,548	22,443
N25 (bp)	2,538	3,369	3,444	4,072	2,185	2,469
N50 (bp)	1,382	1,673	1,682	1,918	1,257	1,307
N75 (bp)	718	812	796	868	680	677

Raw reads were trimmed and low quality and over-represented sequences were removed prior to assembly using Trinity software.

### Annotation of the Reference Transcriptome: BLAST Results and Gene Ontology (GO)

**Figure 4:**
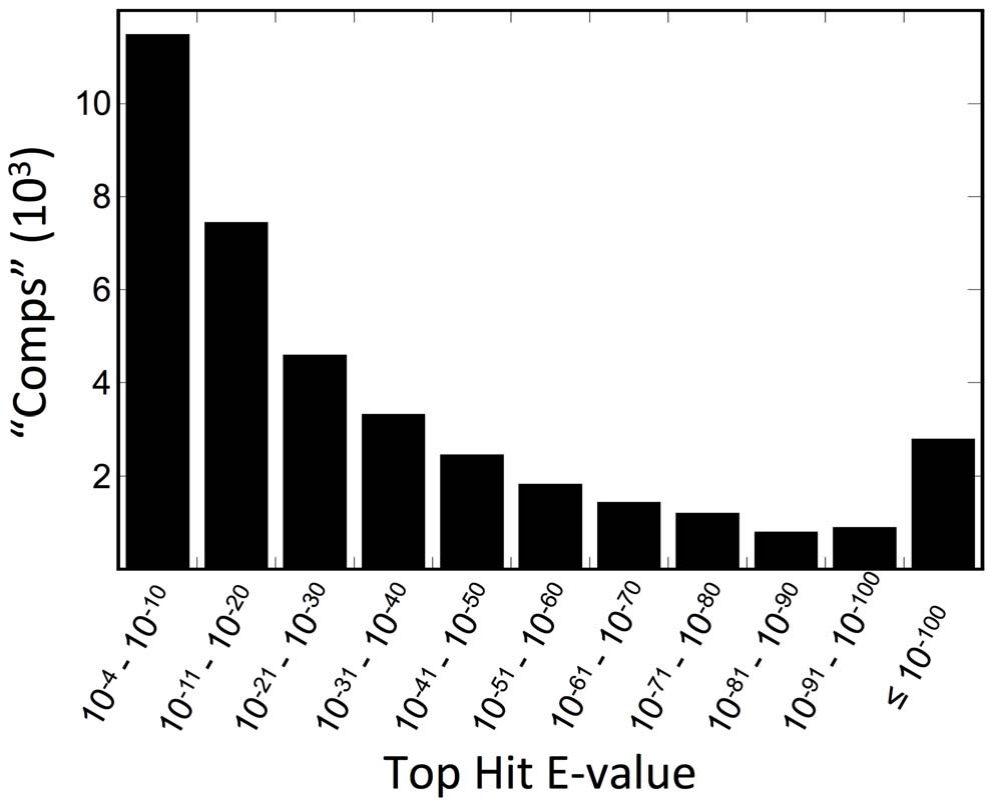
Frequency distribution of best E-values from blastx top hits against nr protein database in NCBI using Blast2GO annotation program for the 96,090-comp reference transcriptome. Search results from February, 2013.

The reference transcriptome, comprising the 96,090 sequences representing unique comps, was annotated using Blast2GO. The assembled sequences were searched against the non-redundant (nr) and SwissProt protein databases using the blastx algorithm with an E-value cutoff set at 10^−3^. Searching against the nr database resulted in 38,289 comps (∼40%) having significant blast hits (Table 5). A large percentage of the comps with no blast hits were short, *i.e.* in the 300–400 bp range (23,403 out of 55,306 sequences). Many of these short sequences probably represent partial transcripts, which may have contributed to the “no blast hit” result. Blastx results using SwissProt as the reference database, which is manually annotated and reviewed, yielded understandably fewer significant hits, comprising 28,616 comps (Table 5). Further analysis for gene ontology using the SwissProt database led to GO and GOSlim annotations of nearly identical numbers of comps, 10,334 and 10,344, respectively (Figure S1). We obtained fewer GO and GOSlim annotations using the nr database as reference (Table 5). Nearly 30% of blastx results against the nr database had top hits with high E-values (>10^−10^), while fewer than 25% had E-values below 10^−50^ (Figure 4). This is consistent with relative paucity of genomic resources for crustaceans [25]. In contrast, blastx homology results of a recent *de novo* transcriptome of an insect, the western tarnished plant bug (*Lygus hesperus*), returned 55% of top hits with E-values below 10^−50^ [30].

**Table 5:** Summary of *Calanus finmarchicus de novo* reference transcriptome annotation statistics using the blastx algorithm with Blast2GO software.

	Nr protein	SwissProt
Total number of sequences	96,090	96,090
Sequences with BLAST matches	38,289	28,616
Sequences with Gene Ontology (GO) terms	5,069	10,334
Sequences annotated with GOSlim	4,632	10,344

Two separate protein databases, non-redundant protein (nr) and SwissProt, were downloaded onto a local computer cluster (Feb. 2013) and searched. Due to a limitation in the blastx software, no transcripts ≥8,000 bp in length were annotated.

Another aspect of the automated annotation is that the blastx algorithm is limited to nucleotide sequences shorter than 8,000 bp. The automated BLAST2GO annotation was not able to process any of the very long comps. Thus, we translated these comps into predicted proteins using an online translation tool (web.expasy.org/translate/?). These translated sequences were manually entered into blastp online and searched against nr protein sequences (http://blast.ncbi.nlm.nih.gov). This led to putative identifications of an additional 130 sequences, which represented expected long transcripts encoding large proteins, such as kettin/titin, supervillin, beta spectrin, midasin, and cytoplasmic dynein 2 heavy chain. Kettin/titin and supervillin are both actin-binding proteins with kettin/titin involved in muscle function. Beta spectrin is a cytoskeletal protein involved in membrane integrity and neuronal function. Midasin is a nuclear chaperone involved in the assembly/disassembly of macromolecules in the nucleus. Cytoplasmic dynein 2 heavy chain is motor protein involved in converting chemical energy (ATP) into mechanical energy (movement). These identifications were added to the reference transcriptome, bringing the total number of comps with significant blast hits to 38,419.

**Figure 5:**
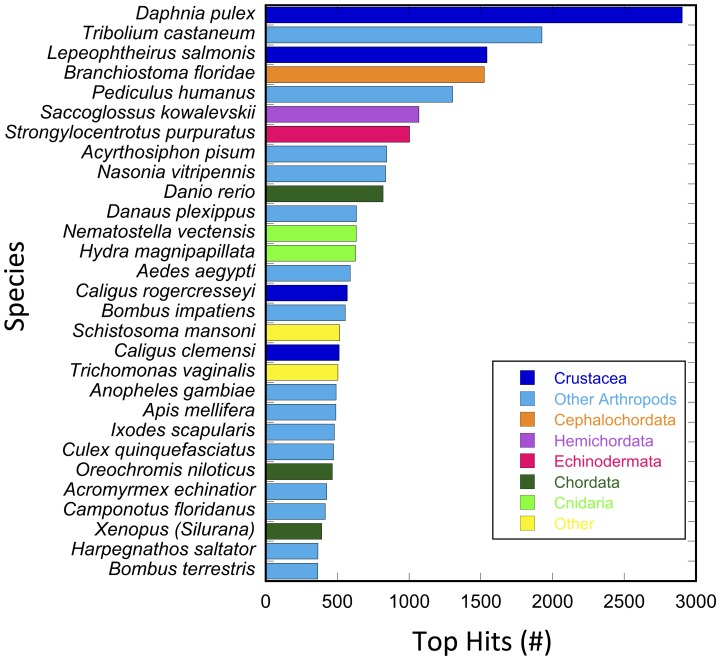
Number of top hits by species from blastx results of searches against nr protein database in NCBI using Blast2GO annotation program for the 96,090-comp reference transcriptome. Taxonomic groups are color-coded: crustaceans (dark blue), other arthropods (light blue), cephalochordates (orange), hemichordates (purple), chordates (dark green), echinoderms (pink), cnidarians (light green) and other (yellow). Search results from February, 2013.

The under-representation of crustaceans with respect to genomic resources was also evident from the taxonomic distribution of top hits in the blastx results for the nr database. Nine taxonomic groups were represented in the 29 top-hit species (Figure 5). Arthropods accounted for only 60% of the species (18 out of 29): four non-malacostracan crustaceans, 13 insects and one chelicerate. The branchiopod crustacean *Daphnia pulex* and the insect *Tribolium castaneum* had the largest and second largest number of top hits (2,905 and 1,927 out of 35,164 hits), respectively. Three parasitic copepod crustaceans (*Lepeophtheirus salmonis*, *Caligus rogercresseyi* and *Caligus clemensi*) were among the top-hit species, with 2,672 combined blast hits with E-values ≤10^−3^. Interestingly, the 29 top-hit species did not include a single *Drosophila* species or any decapod crustaceans. The fourth top-hit species was the lancelet *Branchiostoma floridae* (a cephalochordate), a result that was similar to that found for the amphipod crustacean *Parhyale hawaiensis* by Zeng et al. [25].

**Figure 6:**
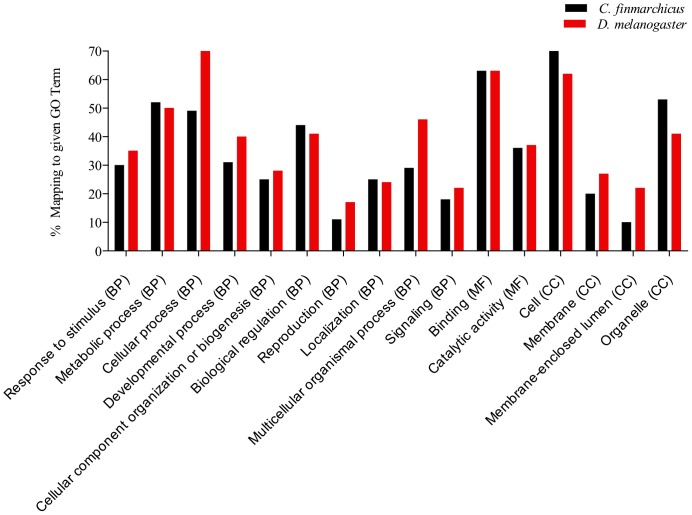
Comparison between the distributions of GO annotations obtained through GOSlim in *Calanus finmarchicus* (black) and *Drosophila melanogaster* (red) for gene ontology level 2 for biological process (BP), molecular function (MF) and cellular component (CC). Percentages were calculated as the number of sequences annotated to a given GO term divided by the total number of GO annotated comps (*C. finmarchicus*) or genes (*D. melanogaster*) (x100). GO annotations for *C. finmarchicus* obtained from searches against SwissProt database. GO annotations for *D. melanogaster* obtained from the annotated genome (http://www.b2gfar.org/showspecies?species=7227). BP: response to stimulus (GO:0050896); metabolic process (GO:0008152); cellular process (GO:0009987); developmental process (GO:0032502); cellular component organization or biogenesis (GO:0071840); biological regulation (GO:0065007); reproduction (GO:0000003); localization (GO:0051179); multicellular organismal process (GO:0032501); signaling (GO:0023052). MF: binding (GO:0005488); catalytic activity (GO:0003824). CC: cell (GO:0005623); membrane (GO:0016020); membrane-enclosed lumen (GO:0031974); organelle (GO:0043226). Blastx searches against SwissProt completed February, 2013.

In order to assess the representation of biological and molecular processes and cellular components among the assembled compounds, the distribution of GO terms in the *C. finmarchicus* transcriptome was compared to that from the genome of *Drosophila melanogaster* (http://www.b2gfar.org/showspecies?species=7227). Broad representation was found in *C. finmarchicus* (Figure 6). At gene ontology level 2, the relative distribution of GO terms was similar to that for the genome of *D. melanogaster*, with the largest proportions of GO annotations for biological process (BP) indicated for involvement in response to stimulus, metabolic, cellular and developmental processes and biological regulation, while binding and catalytic activity were the most common GO annotations for molecular function (MF). Highest percentages in the cellular components (CC) domain were seen in cell and organelle categories. Gene ontology analysis using multi-level pie charts also identified several GO terms that might be indicative of contamination by other organisms. Specifically, we found comps annotated as plastids (GO:0009536), thylacoids (GO:0009579), viral reproduction (GO:0016032) and symbiosis encompassing mutualism through parasitism (GO:0004419). The percentage of reads that mapped to these sequences was between 0.01 and 0.25%. We also searched the annotated sequences for contamination by *Rhodomonas baltica* (the algal food used) and found 14 comps with *Rhodomonas* sp. as top hit species. Mapping of reads against these comps indicated very low contamination and the percentage of mapped reads ranged between 10^−5^ and 10^−6%^. The overall level of contamination in this transcriptome was low.

Previous targeted searches by Christie and colleagues of this *de novo* transcriptome have been performed to identify transcripts of interest involved in neurochemical signaling and circadian rhythms [17], [20], [21]. These searches have been able to confirm the results of the automated annotation, but also led to the identification and more complete annotation of a number of transcripts. In particular, neuropeptides are rarely annotated via automated means and require a targeted workflow for identification [17]. Interestingly, one of the conclusions of these studies has been that the neurochemical signaling systems of *Calanus finmarchicus* appear to be more similar to those of insects than to higher crustaceans, which is supported by our current result of a large number of insects among the top hit species. These results are consistent with the pancrustacean grouping [31]–[33]. However, the position of the copepods within the Pancrustacea is still in question, and this transcriptome may well contribute to the on-going discussions on the phylogenetic placement of copepods.

### Stage-Specific Expression: Global Patterns and Target Transcripts

For an analysis of stage-specific expression patterns, reads from each developmental stage were mapped against the reference transcriptome using Bowtie software. The total number of high quality reads used in the mapping step ranged from 50 million to 66 million reads per stage. Alignment statistics for the individual stages were similar to each other, and to those for all reads combined, suggesting that the Trinity assembly produced similar coverage for all developmental stages (embryo, early nauplius, late nauplius, early copepodite, late copepodite [CV] and adult female). Overall, alignment rate ranged between 73 and 77%, and fewer than 1% of the reads mapped more than once (Table S2).

**Figure 7:**
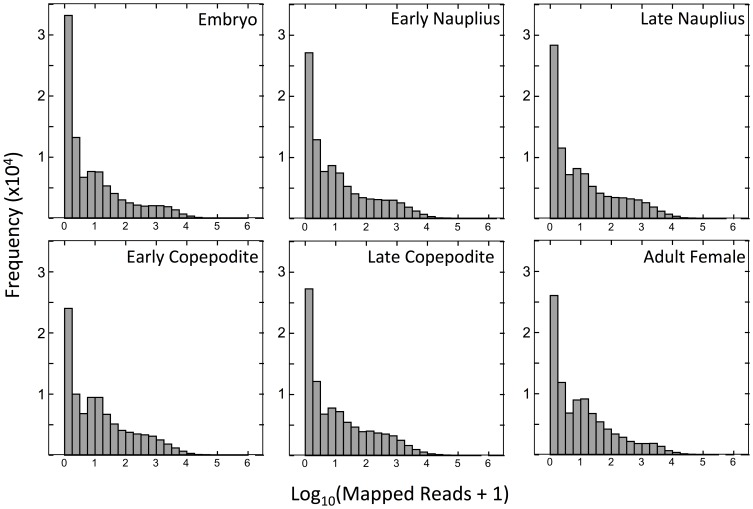
Frequency distribution of the number of mapped reads for each sample on a log scale. Developmental stages: embryo; early nauplius (NI-NII); late nauplius (NV-NVI); early copepodite (CI-CII); late copepodite (CV) and adult female (stage CVI). Trimmed and quality-filtered reads were mapped against the reference transcriptome of 96,090 comps. X-axis intervals are the same as in Figure 2.

Frequency histograms of the number of mapped reads per comp for the six developmental stages were characterized by a large number of comps with very low counts and a broad shoulder with expressions of 10 to1000 mapped reads per transcript (Figure 7). These distributions are in contrast to the frequency histogram of all samples combined (Figure 2). The two lowest bins, representing 0 to 2 mapped reads, have the highest numbers of counts (Figure 7). The percentage of transcripts in these bins ranged between 35 and 48%. Mixed embryos had the largest percentage of sequences with zero reads (34%), while the early copepodites had the lowest (25%). The stage-specific global gene expression data thus suggest that at least 1/3 of the transcriptome was not significantly expressed (compounds with ≤2 mapped reads = ”silent”) during any particular life stage.

**Figure 8:**
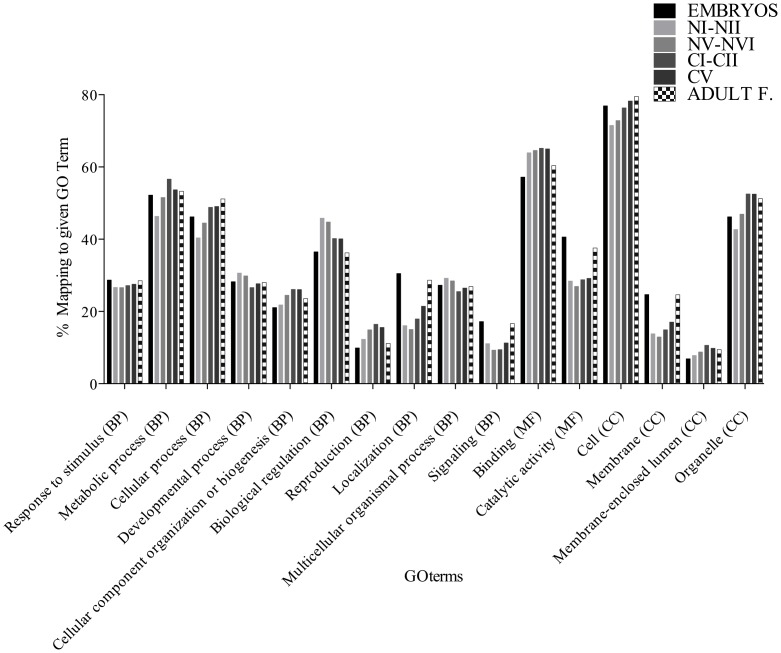
Functional analysis of the unexpressed transcripts (# of mapped reads ≤2) in different developmental stages. Distribution of GO terms through GOSlim at gene ontology level 2 for biological process (BP), molecular function (MF) and cellular component (CC) of annotated comps that were not expressed at each stage. Percentages were calculated as the number of sequences annotated to a given GO term divided by the total number of GO annotated compounds (x100) present in the unexpressed comps. GO terms as in Figure 6. Total number of unexpressed transcripts with GO terms: embryo (2,933), early nauplius (1,777), late nauplius (1,900), early copepodite (2,227), late copepodite (2,501) and adult female (2,463).

In order to gain some insight into the function of the silent transcripts, we analyzed their gene ontology. Of the 10,344 GO-annotated comps, the number of silent transcripts in any one stage ranged from 1,777 to 2,933. For each developmental stage, the relative abundance of silent transcripts by function at gene ontology level 2 is shown in Figure 8. These transcripts represented a wide range of biological processes, molecular function and cellular component. In many categories, the proportion of transcripts annotated was similar across stages with percentages differing by less than 5%. The exception to this was the biological process localization (GO:0051179), which was under-represented in nauplii and over-represented in embryos and adult females. The percent of membrane proteins (CC, GO: 0016020) was also over-represented in embryos and adult females, and a similar pattern was observed for catalytic activity (MF, GO:0003824). At this level of organization, this analysis only provided limited insights into the function of the unexpressed transcripts.

For a more detailed analysis of stage-specific expression patterns, we examined transcripts involved in lipid biosynthesis. Although it is well known that fatty acids are critical for normal development, the regulation of lipid metabolism is still poorly understood in eukaryotes, and is an active area of research [34]. In *C*. *finmarchicus*, lipid stores play an important role in the life history, including growth, reproduction, resistance to starvation and timing of pre-adult diapause [35], [36]. Although lipid stores have been quantified in this species [37], less is known about the specific pathways and their regulation in different life stages and/or under environmental fluctuations. The annotated reference transcriptome was searched for compounds putatively encoding proteins that are involved in the establishment of lipid stores using as a guide the Kyoto Encyclopedia of Genes and Genomes (KEGG; http://www.genome.jp/kegg/pathway.html) list of lipid biosynthesis proteins found in *D. melanogaster*. Thirty-seven compounds with significant expression (# of mapped reads) were so identified (Table 6). Among these putative identifications were seventeen acyltransferases, eleven elongases and three desaturases. Searches of the extant *C. finmarchicus* EST database using the identified compounds as the query sequences provided strong support (≥95% identity at the nucleotide level in regions of overlap) for 10 transcripts (footnotes in Table 6). However, many of these ESTs had not been annotated, so this is the first extensive list of putative transcripts involved in lipid biosynthesis for this species.

**Table 6:** Comps encoding predicted transcripts involved in lipid biosynthesis as annotated by Blast2GO.

Compound	Annotation	E.C. number	L (bp)	Top Hit species	Accession #	E-value
Fatty Acid Synthase						
comp211898	palmitoyl-protein thioesterase 1	3.1.2.22	1103	*Acyrthosiphon pisum*	XP_001951992	1.79E-58
comp221683	3-hydroxyacyl-CoA dehydratase 2	1.1.1.35	825	*Ascaris suum*	ADY48993	3.08E-45
comp374560^1^	3-oxoacyl-acyl-carrier-protein synthase mitochondrial	2.3.1.41	1425	*Camponotus floridanus*	EFN63787	7.24E-109
comp716955	3-oxoacyl-acyl-carrier-protein reductase	1.1.1.100	994	*Lepeophtheirus salmonis*	ADD38528	2.18E-65
comp915320	acyl-coenzyme a thioesterase mitochondrial	3.1.2.2	825	*Ascaris suum*	ADY48993	3.08E-45
Desaturase						
comp5606^2^	delta-9 desaturase	1.14.19.1	1406	*Ctenopharyngodon idella*	CAB53008	3.54E-92
comp718898	stearoyl-CoA desaturase (delta-9-desaturase)	1.14.19.1	1173	*Xenopus laevis*	NP_001087809	1.96E-74
comp546275	delta-6 fatty acid desaturase	1.14.19.-	1112	*Caligus rogercresseyi*	ACO10922	4.42E-89
Elongase						
comp35445^3^	elongation of very long chain fatty acids protein AAEL008004	2.3.1.199	985	*Tribolium castaneum*	XP_968784	3.00E-67
comp71231^4^	elongation of very long chain fatty acids protein AAEL008004	2.3.1.199	1387	*Apis mellifera*	XP_623221	3.72E-78
comp139521	elongation of very long chain fatty acids protein AAEL008004	2.3.1.199	1544	*Nasonia vitripennis*	XP_001600048	2.91E-66
comp25729	elongation of very long chain fatty acids protein AAEL008004	2.3.1.199	937	*Acyrthosiphon pisum*	XP_001952818	8.00E-59
comp140341	elongation of very long chain fatty acids protein AAEL008004	2.3.1.199	1095	*Tribolium castaneum*	XP_968784	2.73E-75
comp100109_	elongation of very long chain fatty acids protein AAEL008004	2.3.1.199	759	*Tribolium castaneum*	XP_968784	9.99E-21
comp139813	elongation of very long chain fatty acids protein AAEL008004	2.3.1.199	760	*Bombus terrestris*	XP_003399502	9.92E-45
comp767531	elongation of very long chain fatty acids protein AAEL008004	2.3.1.199	820	*Tribolium castaneum*	XP_968784	3.46E-57
comp854733	elongation of very long chain fatty acids protein AAEL008004	2.3.1.199	360	*Bombus terrestris*	XP_003399502	1.72E-15
comp82975	elongation of very long chain fatty acids protein 6-like	2.3.1.199	1501	*Bombus terrestris*	XP_003401825	2.76E-98
comp296905	elongation of very long chain fatty acids protein 4-like	2.3.1.199	1239	*Apis mellifera*	XP_395160	8.60E-84
Acyl-CoA synthetase						
comp65215	fatty acid binding protein		569		3PPT_A	1.84E-15
Phospholipid acyltransferase						
comp73859^5^	glycerol-3-phosphate acyltransferase 3-like isoform 3	2.3.1.15	2017	*Nasonia vitripennis*	XP_003424470	3.18E-143
comp248238	glycerol-3-phosphate acyltransferase 3-like isoform 3	2.3.1.15	1628	*Nasonia vitripennis*	XP_003424470	2.13E-123
comp432493	glycerol-3-phosphate acyltransferase 4-like	2.3.1.15	422	*Acromyrmex echinatior*	EGI59997	1.52E-35
comp15342^6^	dihydrolipoamide branched chain transacylase e2	2.3.1.168	1755	*Danio rerio*	NP_001013533	2.26E-134
comp27780^7^	diacylglycerol o-acyltransferase	2.3.1.20	1481	*Pediculus humanus corporis*	XP_002430154	2.11E-50
comp342802	2-acylglycerol o-acyltransferase 1	2.3.1.20	1139	*Caligus rogercresseyi*	ACO11005	1.80E-101
comp220465	lysophospholipid acyltransferase lpcat4	2.3.1.23	1236	*Anolis carolinensis*	XP_003223902	6.41E-47
comp46978^8^	lysophosphatidylcholine acyltransferase 1 lpcat1_2	2.3.1.23; 2.3.1.67	2136	*Nasonia vitripennis*	XP_001603929	1.78E-75
comp70420^9^	lysophosphatidylcholine acyltransferase 2 lpcat1_2	2.3.1.23; 2.3.1.67	2484	*Nasonia vitripennis*	XP_001603929	6.48E-56
comp873201	lysophosphatidylcholine acyltransferase 2 lpcat1_2	2.3.1.23; 2.3.1.67	1278	*Gallus gallus*	NP_001025739	3.04E-39
comp66316	1-acyl-sn-glycerol-3-phosphate acyltransferase alpha	2.3.1.51	920	*Danaus plexippus*	EHJ76952	3.41E-46
comp202171	1-acyl-sn-glycerol-3-phosphate acyltransferase gamma	2.3.1.51	1437	*Harpegnathos saltator*	EFN82046	6.70E-70
comp349956	1-acyl-sn-glycerol-3-phosphate acyltransferase gamma-like	2.3.1.51	1497	*Nasonia vitripennis*	XP_001607215	6.85E-65
comp86721	1-acylglycerol-3-phosphate acyltransferase	2.3.1.51	1338	*Culex quinquefasciatus*	XP_001849976	3.10E-74
comp384379^10^	lysocardiolipin acyltransferase 1 lclat1	2.3.1.51; 2.3.1.-	1837	*Nasonia vitripennis*	XP_001603121	4.60E-61
comp128361	acyl-CoA :lysophosphatidylglycerol acyltransferase 1-like	2.3.1.-	1201	*Lepeophtheirus salmonis*	ADD38123	3.34E-85
comp137040	acetyl-Coenzyme A acyltransferase 2-like	2.3.1.9	1567	*Saccoglossus kowalevskii*	XP_002732194	6.92E-132

1 comp374560: EL773889 (95%).

2 comp5606: EL965872 (95%); EH666911 (95%).

3 comp35445: ES387246 (96%); ES387223 (96%).

4 comp71231: FG342192 (99%).

5 comp73859: GR410954 (98%).

6 comp15342: FK671214 (98%); FK671000 (96%); FK671010 (95%).

7 comp27780: FK670374 (97%); FG632581 (97%); FG342908 (97%).

8 comp46978: EL697137 (99%); EL965732 (99%).

9 comp70420: EL773351 (95%).

10 comp384379: FG342951 (97%).

The list excludes 18 compounds with very low expression levels (<100 reads per transcript for all samples combined). E.C.: enzyme commission number. Comps with superscript numbers indicate EST support. EST accession numbers and percent identity to their respective comp sequence are given in the footnotes.

**Figure 9:**
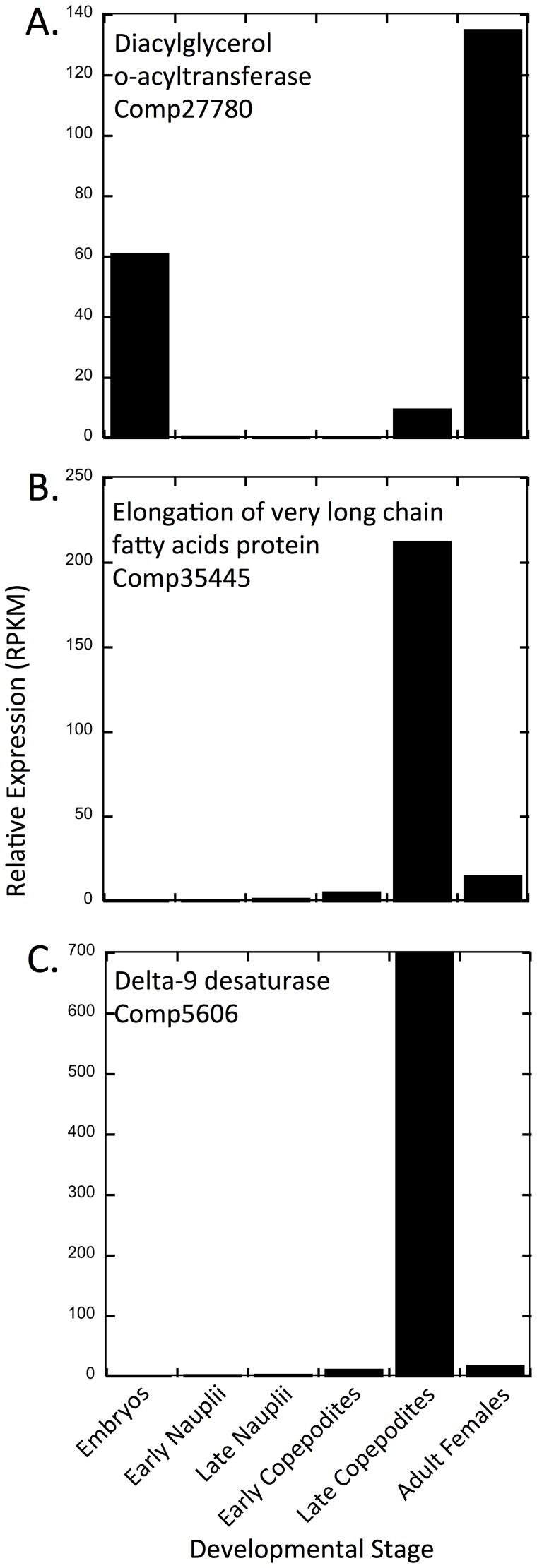
Relative expression of three transcripts involved in lipid biosynthesis in different stages. Relative expression presented as RPKM (reads per kilobase per million mapped reads). A. Diacylglycerol o-acyltransferase (comp27780). B. Elongation of very long chain fatty acids protein (comp35445). C. Delta-9 desaturase (comp5606).

Pronounced stage-specific expression was observed in three lipid metabolism transcripts, one acyltransferase, one elongase and one desaturase (Figure 9). These three transcripts were highly expressed in either one (elongase, desaturase) or two (acyltransferase) developmental stages. Expression levels differed by more than 100-fold between the highest and lowest stages. The most highly expressed acyltransferase (comp27780) transcript was identified as diacylglycerol o-acyltransferase 1, which is involved in the last step of triglyceride ( = triacylglycerol, TAG) synthesis in eukaryotes [38]. This transcript had partial overlap with three *C. finmarchicus* ESTs (Accession Nos. **FK670374**, **FG632581**, **FK867624**). The highest expression levels of this transcript were observed in adult females (>100 RPKM) and in embryos (∼ 60 RPKM), with moderate/low levels seen in late copepodites (stage CV, ∼ 9 RPKM; Table 7). Expression in all other stages was below 1 RPKM (Figure 9A). This expression pattern is consistent with lipid storage associated with egg production. Three other acyltransferases (comp66316, comp70420, comp73859) showed peak expression in embryos and adult females (20 to 40 RPKM, Table 7). Other expression patterns with developmental stages among the acyltransferases included similar expression across all samples, or broad expression peaks across three to four stages (Table 7).

**Table 7:** Relative expression of transcripts identified as involved in lipid biosynthesis.

	Function	Embryo	Early Nauplius	Late Nauplius	Early Copepodite	Late Copepodite	Adult Female
comp15342	PLAT	**26.3**	**43.2**	**26.2**	**29.8**	21.3	**23.8**
comp86721	PLAT	**16.5**	**9.4**	**11.4**	**9.8**	**15.9**	**18.6**
comp128361	PLAT	**12.9**	**14.4**	**15.1**	**12.1**	**14.6**	**15.1**
comp342802	PLAT	0	**8.3**	**5.3**	**8.4**	**6.1**	0.6
comp349956	PLAT	1.4	**6.3**	**4.5**	**6.3**	**7.3**	3.4
comp384379	PLAT	0.9	**6.3**	**6.5**	**4.3**	**4.7**	1.0
comp221683	FAS	3.5	**8.7**	**9.5**	**13.0**	**8.5**	5.7
comp211898	FAS	0.2	**9.5**	**9.5**	**13.6**	6.4	1.0
comp137040	PLAT	5.7	**19.7**	**13.3**	**12.0**	5.9	2.0
comp100109	Elo	0.1	**16.0**	**19.8**	**19.1**	2.4	1.4
comp139813	Elo	0.2	**16.5**	**15.3**	**11.2**	5.4	4.8
comp82975	Elo	0.4	**10.6**	**13.2**	**19.8**	9.5	8.9
comp296905	Elo	1.9	**6.9**	**8.6**	**6.2**	1.7	0.3
comp140341	Elo	0.3	**12.8**	**11.9**	**17.4**	7.8	1.5
comp718898	Des	0	1.6	**3.1**	**5.0**	1.5	1.8
comp248238	PLAT	0.1	0.3	0.4	**3.0**	**4.0**	0.2
comp202171	PLAT	0.8	3.4	5.4	**12.8**	**8.0**	3.2
comp65215	ACoA	0.7	20.1	**49.5**	13.3	17.4	0
comp546275	Des	<0.1	2.6	3.8	**8.1**	3.6	0.5
comp5606	Des	0.3	1.8	2.6	10.8	**699**	17.3
comp35445	Elo	0.2	0.7	1.5	5.3	**212**	14.8
comp25729	Elo	0.1	8.7	9.9	7.9	**35.7**	4.2
comp66316	PLAT	19.9	10.3	6.1	8.0	17.2	**42.7**
comp27780	PLAT	60.8	0.5	<0.1	0.3	9.4	**135**
comp70420	PLAT	**33.2**	10.0	6.6	8.7	5.7	15.4
comp73859	PLAT	**20.9**	11.9	9.9	12.2	11.8	**36.7**
comp374560	FAS	**8.7**	3.7	3.3	3.4	3.1	**7.8**
comp46978	PLAT	**37.9**	**20.4**	18.2	17.0	15.9	**33.1**
comp220465	PLAT	3.3	**10.2**	**7.3**	7.1	**14.4**	**10.5**
comp71231	Elo	0.4	5.8	**19.3**	15.3	**31.6**	12.7
comp767531	Elo	0	0.5	**3.0**	**4.8**	1.0	**3.2**

Expression levels are given in reads per kilobase per million (RPKM). Bolded values have mapped reads in excess of 50% of the maximum expression for all stages for that comp. Abbreviations for function: fatty acid synthase (FAS); desaturase (Des); elongase (Elo); acyl-CoA sythetase (ACoA); and phospholipid acyltransferase (PLAT).

The other two transcripts showed even more extreme patterns with expression levels in late copepodites (stage CV) at 200 and 700 RPKM, respectively (Figure 9B,C). One of these transcripts, comp35445 was annotated as an elongase. Lipid-rich stage CV copepodites collected in the Gulf of Maine in July are presumably preparing to enter diapause [9], [37], [39]. This requires the synthesis of wax esters, formed from fatty acids largely derived from the diet, and esterified with fatty alcohols synthesized *de novo*, the latter step involving chain elongation [40]. This elongase had support from two non-overlapping ESTs (ELOV4: Accession No. **ES387246**, and unidentified: **ES387223**), which had been identified in a previous study as up-regulated in shallow (actively preparing for diapause) vs. deep (presumably diapausing) pre-adult *C. finmarchicus* [9]. Our results are consistent with the pattern of up-regulation described by Tarrant using subtractive hybridization [9]. In addition, our data suggest that this elongase is highly expressed in the pre-adult stage, but not in the other developmental stages examined here. Thus, this elongase may be involved in the regulation of diapause, as it catalyzes the synthesis of very long chain fatty acids, which, given the timing of its expression, would likely contribute to an increase in total lipid storage in sub-adult individuals preparing to enter diapause. A similar up-regulation of elongase expression, presumably also in preparation for diapause, has been noted in several insect species [41]–[43].

The expression pattern seen for the comp35445 elongase was not representative of the other elongases (Table 7). Although a common feature among the elongases was low expression in embryos, expression patterns varied among comps. Peak expression in the late copepodite was observed in two other elongases (comp25729 and comp71231), but overall expression was lower and expression differences were not as extreme across developmental stages (Table 7). Five elongases showed high expression in multiple developmental stages, in particular in the nauplii (early and late) and early copepodites (Table 7). These expression patterns suggest that the elongases differ from each other in function, and each may play a very specific role during development. Recent studies on model organisms have focused on stage-specificity in expression of transcripts and proteins involved in lipid metabolism, and have started to reveal that elongases can have very specific roles during development [34].

The putative delta-9 desaturase (comp5606) had very high expression in the late copepodites (CV). Comp5606, which encodes a full-length protein, had support from two EST sequences encoding partial proteins (Accession Nos. **EH666911** and **EL965872**). In contrast, the two other putative desaturases (comp546275 and comp718898) had much lower expression and their peak expression was observed in the early copepodites with RPKM values of less than 10 (Table 7). Delta-9 desaturase is the rate-limiting enzyme in the formation of monounsaturated fatty acids from saturated fatty acids, and thus is a critical component in the production of phospholipids, triglycerides and cholesteryl esters, the synthesis of which is accomplished using its products. It is involved in the *de novo* biosynthesis of the fatty alcohol component of wax esters [40]. As with the putative elongase encoded by comp35445, the timing of expression of this delta-9 desaturase is consistent with it contributing to an increase in total lipid storage in CV individuals preparing to enter diapause, a function previously shown for homologous transcripts/proteins in diapausing insects [41]–[43].

These expression results of the delta-9 desaturase, elongase, and the acyltransferase are interesting and they present a marked contrast of wax ester vs. triacylglyceride biosynthesis between the late copepodite (CV) and adult. Furthermore, given the number of sequences identified with similar functional annotations, the transcripts involved in these processes may be very specific, and potentially are good biomarkers for physiological processes.

### Significance of Multiplicity of Sequences in Complete Transcriptome

23% of the comps (22,165 out of 96,090) contained clusters of multiple contigs. Potential sources of sequence variation that might combine multiple contigs into single “comps” include polymorphisms within a population, presence of two or more genes of recent origin from a common source, alternative splicing and differences in the targeting signals, leading to differences in the untranslated regions (UTR) [14]. Thus, these types of sequence variants potentially have a biological source that must be considered in transcriptome analyses. However, some variability in sequences may also arise from assembly artifacts. In order to examine how different factors contributed to sequence multiplicity within compounds in our assembly, we examined transcripts putatively encoding the alpha subunit of voltage-gated sodium channels (Na_V_). The principal unit of the Na_V_ is a large protein with four repeat domains (R1–R4) of six trans-membrane segments each (S1–S6). These are highly conserved and have been well studied in model systems [44]–[46]. In *C. finmarchicus*, these channels are potential targets for saxitoxin originating from seasonal blooms of the dinoflagellate *Alexandrium fundyensis*.

Multiplicity of function in the voltage-gated sodium channel has been achieved in various taxa both through mechanisms producing splice variants and through gene duplication. The former has been described for *D. melanogaster*, while the latter has been found in vertebrate model organisms [44], [45]. However, it is not clear how many different voltage-gated sodium channels are present in crustaceans. Currently, there are few annotated Na_V_ sequences available for crustaceans through NCBI; a search conducted on 6/24/2013 identified just two full-length sequences (*Cancer borealis* and *Daphnia pulex*) and three partial ones (*Lepeophtheirus salmonis, Hyalella azteca*, *Scylla paramamosain*). In *D. melanogaster*, two genes (Na_V_1 and Na_V_2) encoding voltage-gated sodium channels have been identified in the genome [46]. The Na_V_1 protein is also known as *paralytic* or *para*, and it has been shown to include multiple isoforms, many resulting from alternative splicing [47], [48].

The *D. melanogaster* Na_V_1 channel, *para* isoform A protein sequence (Accession No. **P35500**) was used as a query to identify similar sequences in the *Calanus* transcriptome. Five comps were identified as highly similar with E-values below 10^−100^ (Na_V_1-I to Na_V_1-V; Table 8). Each comp was represented by multiple contigs ranging in number from two to 20. One comp (comp44060) included two Butterfly disconnected subgraph groups (“c” groups) with two contigs each, while the rest were assigned to a single such group (Table 8). A reciprocal blast against the nr arthropod database using the blastp algorithm indicated that all contigs but two were most similar to insect *para* sodium channels with top hits having E-values of 0.0 (Table 9). The two contigs (comp44060_c0_seq1 and comp44060_c0 _seq2) that did not identify a *para* sodium channel as a top hit were removed from all further analysis. A *D. melanogaster* Na_V_2 channel protein sequence (Accession No. **Q9W0Y8**) was used to query the complete transcriptome for putative Na_V_2 transcripts. A single comp with three contigs was identified (Table 8). A reverse blast against all nr arthropod proteins identified the sequence as most similar the sodium channel 60E (an alternative designation for an Na_V_2) from an ant (E-value = 0.0; Table 9). Seven additional comps were identified as voltage-gated sodium channels by searching the “top hits” in the Blast2GO annotations (Table 8). These were shorter in length, had higher E-values, and all except one consisted of single contigs. These comps appeared to comprise contigs encoding portions of the Na_V_1 channel (Table 8,9). One additional short sequence (670 bp) putatively encoding a Na_V_2 channel was not included in this list, since there were questions regarding its correct identification given a high E-value (1.4×10^−6^).

**Table 8:** Identification of putative voltage-gated sodium channels in the *Calanus finmarchicus de novo* transcriptome using *Drosophila melanogaster* sequences as queries in blastx searches (completed prior to August 1, 2013).

Name	Comp ID	# contigs	Longest contig		Translated Region
			Nucleotide length (bp)	Predicted protein length (aa)	
Calfi- Na_V_1-I	comp222993_c0	20	6892	1632	RII to C-terminal
Calfi- Na_V_1-II	comp44060_c3	2	7783	1677	RII to C-terminal
Calfi- Na_V_1-III	comp682803_c1	5	6618	2036	Full length
Calfi- Na_V_1-IV	comp299307_c0	2	2259	483	N-terminal to RI
Calfi- Na_V_1-V	comp233807_c0	3	2701	559	N-terminal to RI
Calfi- Na_V_2	comp428211_c0	3	7631	2475	Full length
Short Sequences	comp3170389_c0	1	379	126	RI-S4 to RI-P
	comp2882012_c0	1	309	102	RI-P to RI-S6
	comp1944448_c0	1	1391	463	R-L to RII-S4
	comp2429191_c0	1	931	310	RI-L to RII-S2
	comp4062604_c0	1	524	135	RIII-P
	comp1578036_c0	2	943	314	RIII-L to RIV-S6
	comp2592041_c0	1	1016	202	RIV-L

Translated region key: R = repeat I - IV; S = transmembrane segment 1–6; L = cytoplasmic loop following a repeat; P = “P-loop” between S5 and S6.

**Table 9:** Reciprocal blastp analyses of putative *Calanus finmarchicus* voltage-gated sodium channels identified in Table 8 against all NCBI curated non-redundant arthropod proteins (searches completed prior to August 1, 2013).

Name	Comp ID	Accession Number	Species	Protein description	BLAST score	E-value
Calfi-Na_V_1-I	comp222993_c0	ACB37024	*Aedes aegypti*	voltage-gated para-like sodium channel	1768	0.0
Calfi-Na_V_1-II	comp44060_c3	AAC47484	*Blatella germanica*	para sodium channel	1502	0.0
Calfi-Na_V_1-III	comp682803_c1	NP_001159381	*Tribolium castaneum*	paralytic B	1788	0.0
Calfi-Na_V_1-IV	comp299307_c0	NP_001188650	*Drosophila melanogaster*	paralytic, isoform BC	657	0.0
Calfi-Na_V_1-V	comp233807_c0	NP_001188650	*Drosophila melanogaster*	paralytic, isoform BC	553	0.0
Calfi- Na_V_2	comp428211_c0	EGI69876	*Acromyrmex echinatior*	sodium channel 60E	1895	0.0
Short contigs	comp3170389_c0	NP_001128390	*Nasonia vitripennis*	voltage-gated sodium channel alpha subunit	171	5.4E-41
	comp2882012_c0	AAD22957	*Leptinotarsa decemlineata*	voltage-gated sodium channel alpha subunit	159	2.7E-37
	comp1944448_c0	BAF37094.2	*Plutella xylostella*	voltage-gated sodium channel alpha subunit splicing variant 2	268	5.4E-69
	comp2429191_c0	ACV87001	*Bombyx mori*	voltage-sensitive sodium channel	139	2.0E-30
	comp4062604_c0	ACB37024	*Aedes aegypti*	voltage-gated sodium channel alpha subunit	84	2.7E-14
	comp1578036_c0	EFA11554.1	*Tribolium castaneum*	voltage-gated sodium channel alpha subunit	421	2.1E-115
	comp2592041_c0	XP_002427248	*Pediculus humanus corpori*	voltage-gated sodium channel	208	5.4E-51

**Figure 10:**
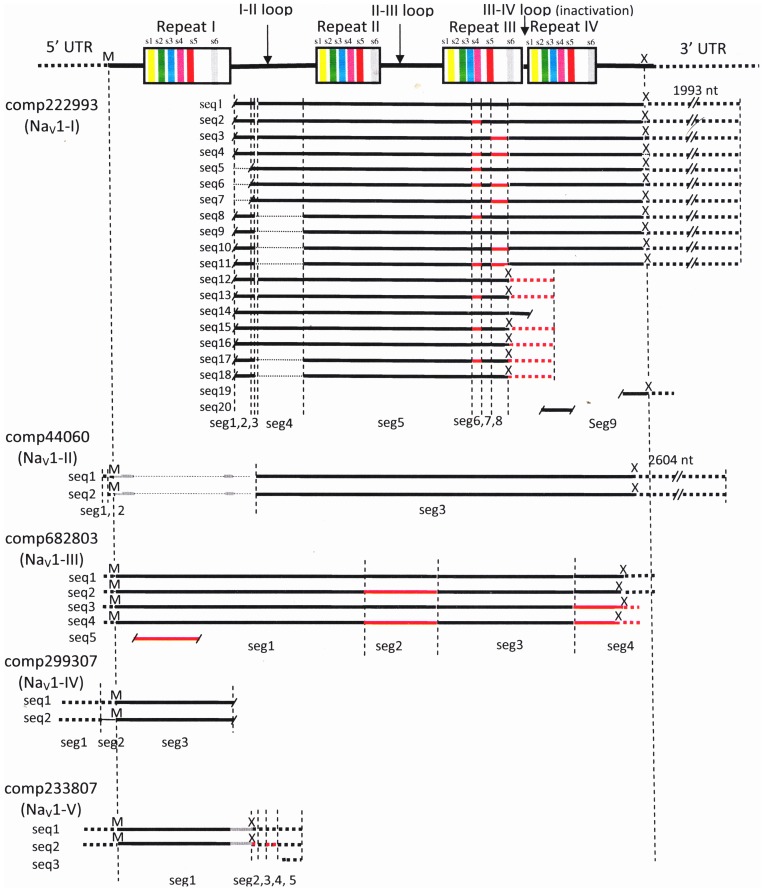
Summary of overall alignment of multiple comps and contigs from the *de novo* assembly identified as Na_V_1 using a *D. melanogaster* Na_V_1 sequence as query. Alignment of translated sequences shown, UTR regions are indicated with dotted lines. Locations of the four transmembrane regions are shown in the top schematic for the *D. melanogaster* query. For each comp, contigs are partitioned into segments (“seg”) to represent regions that differed among the sequences, and putative alternatively spliced segments are shown in red. Discontinuous lines indicate that segments were missing from some sequences. “M” indicates start codon locations and “X” stop codons. Scale for sequence lengths approximate. Comp44060 only represents contigs classified as “c3” by Trinity.

Only a single Na_V_1 sodium channel gene has been identified in the *D. melanogaster* genome [46], and indeed we can only identify one such in the NCBI databases for *Daphnia* (accession **EFX81393**). Thus, the question arises as to whether the different comps identified as Na_V_1 represented transcripts derived from a single gene or whether it was more likely that they resulted from multiple genes. To assess this, the longest sequence from each comp was translated and aligned using the online program MAFFT. These alignments confirmed significant differences among the Na_V_1 sequences. One comp (Na_V_1-III [comp682803]) appeared to code a full-length sodium channel protein and included all four conserved subunit repeats of the 6 membrane-spanning segments (Figure 10). Two (Na_V_1-IV [comp299307] and Na_V_1-V [comp233807]) seemed to be partial N-terminal sequences, while the other two (Na_V_1-I [comp222993] and Na_V_-II [comp44060_c3]) represented partial C-terminal sequences (Figure 10). The two pairs of overlapping comps differed substantially in their amino acid sequences both from each other and from homologous regions of Na_V_1-III (comp682803). Identity across each of the four conserved repeat domains of the comps was modest, ranging from 57% to 87%, suggesting that they represented transcripts from different genes. However, the N- and C-terminal partial sequences seem likely to represent two parts of single transcripts, Na_V_1-I and IV being the most *Drosophila*-like (76–83% identity in conserved domains) and Na_V_1-II and V being less so (64–68% identity). Thus, the five longest compounds assembled by Trinity represent transcripts from putative *para* sodium channels (Na_V_1) that appeared to have been derived from three different genes. Alignment of the remaining seven Na_V_1 compounds obtained from the Blast2GO annotations indicated that these sequences encoded different portions of the protein, with minimal overlap with each other (Table 8). All of these sequences differed from Na_V_1-I to V in corresponding regions, even in conserved transmembrane regions, albeit portions were up to 90% identical. Thus, these short sequences may represent partial assemblies of a fourth Na_V_1 channel. The occurrence of genes split among different comps is not surprising given 96,090 comps in an organism expected to have closer to 20,000 genes. It presumably reflects the incompleteness as well as the statistical limitations of such assemblies.

All Na_V_1 sequences adhered to the DEKA pattern (Asp, Glu, Lys, Ala) in the 4-amino-acid ring forming the selectivity filter that characterizes Na_V_1 channels [49], [50], albeit the total of 40 amino acids of the 4 pore-lining regions (p-loops) differed from the homologous regions of *D. melanogaster* by 2, 4 or 8 residues respectively for putative genes Na_V_1-I&IV, II&V and III. The differences with respect to the *Daphnia* Na_V_1 (accession **EFX81321**) were greater (7, 8 and 11, respectively). If confirmed, this represents, to our knowledge, the first evidence for multiplicity within the Na_V_1 family in a protostome.

In contrast to the *para* channel, a single comp encoding a putative full-length Na_V_2 channel was identified (Table 8,9). This had a selectivity filter pattern of DEEA, diagnostic for Na_V_2 of *Drosophila* and other protostomes [49]. The aggregate p-loops differed by 3 residues from *Drosophila*, compared with 5 in *Daphnia* Na_V_2 (accession EFX89321).

Another question concerns the multiplicity of sequences within each comp. Alternative splicing and intra-sequence deletions have been documented in *D. melanogaster*, which may have as many as 19 functional isoforms of the Na_V_1 gene with each producing pharmacologically distinct sodium channels [51]. Trinity is designed to collect such variants into clusters [14]. In order to obtain some insights into the diversity of Na_V_ channels in *C. finmarchicus*, we aligned the multiple contigs within each comp and analyzed their patterns.

Occurrence of *splice variants* would be one source of extensive sequence differences clustered or restricted to specific parts of the molecule. We searched for such clustering by dividing the nucleotide sequences within one comp into “segments,” with the boundaries defined by transitions in either direction between matched and unmatched in comparisons across sequences (*e.g.* sequence divergence, convergence or termination points). Within-comp differences tended to be grouped and specific to a given region or a few regions. Predicted protein sequences of the five transcripts coding for Na_V_1-III include two sequence variants (“substitutions”) each in segments 2 and 4 suggesting alternative splicing (Figure 10). However, the most extensive example of clustered-variation was found within the Na_V_1-I compound, where two and only two variants were found in each of segments 6, 8 and 9, and these occurred in 7 of the 9 possible combinations (Figure 10).

Intra-sequence insertion/deletion patterns, including those occurring at the ends also contributed to observed sequence variation among contigs within one comp. Deletions typically produce variation in length unless compensatory insertions occur elsewhere in the remaining sequence. This source of variation was observed in Na_V_1-II and Na_V_1-IV, and it was particularly prominent in Na_V_1-I with segments 1, 3 and 4 absent in various combinations with the sequence variants in segments 6, 8 and 9 (Figure 10). Premature termination of the expected amino acid sequence was observed in Na_V_1-I, where mid-sequence “stop” codons produced predicted partial proteins for contigs 12–18 (excluding 14). This truncated the sequence at segment S6 of the 3^rd^ repeat. Similar premature terminations were found in Na_V_1-V, in the Na_V_1 fragment comp4062624 and in the Na_V_2 fragment comp4340636. This type of termination, leading to truncated channel proteins, has been reported in several taxa and may be presumed to serve an as-yet undetermined biological function [51].

There was some evidence for assembly errors in at least one set of contigs. Specifically, we found that in one case two parts of valid sequences appeared to have been joined to form a “chimera”. The translated Na_V_1-II (comp44060_c3), when compared to *Drosophila* Na_V_1, consisted of an N-terminal piece that blasted as complexin, and a non-contiguous C-terminal portion that showed good alignment with the Na_V_1 *D. melanogaster* sequence (Figure 10). Between these two regions was a stretch ∼450 aa in length only sparsely assigned matches by MAFFT (indicated by a thin line in Figure 10).

The picture emerging from these studies, awaiting confirmation from conventional molecular approaches, is that *Calanus finmarchicus* possesses at least four voltage-gated sodium channel genes, one of which is a protostome “Na_V_2” channel and the other three of which represent members of a family of Na_V_’s similar to, though less numerous than, the vertebrate Na_V_1 family. Splice variants are present in at least two members of this latter family, and one of these in particular is expanded into a large family of splice variants, similar to the situation found in *D. melanogaster*.

**Figure 11:**
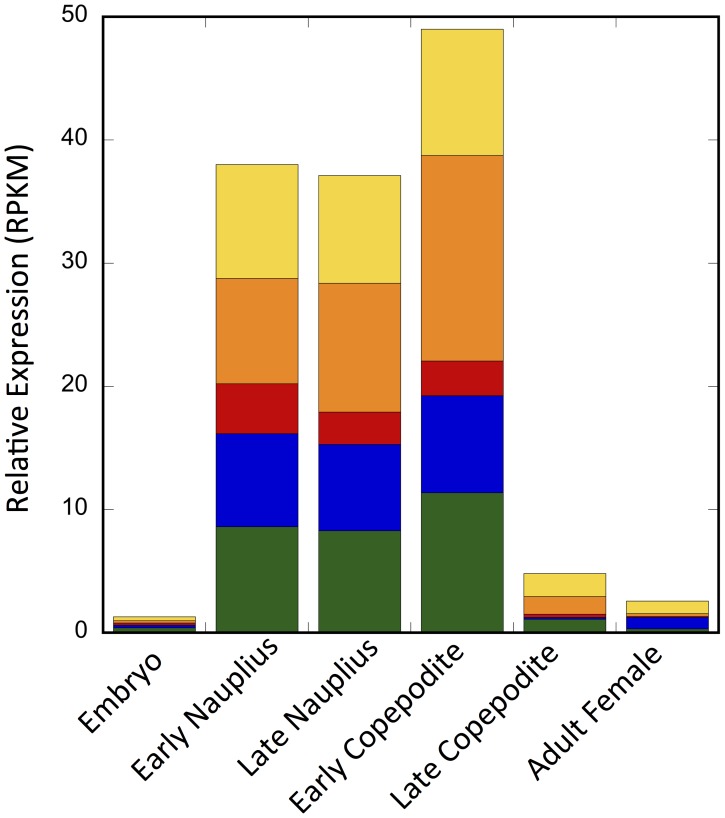
Relative expression of putative Na_V_1 transcripts for different developmental stages. Yellow: Na_V_1-I (comp222993); orange: Na_V_1-II (comp44060_c3); red: Na_V_1-III (comp682803); blue: Na_V_1-IV (comp299307); green: Na_V_1-V (comp428211).

Relative expression of Na_V_ transcripts among developmental stages showed a similar pattern for both Na_V_1 and Na_V_2 sequences: low expression in the embryo, adult females and late copepodites (CV), and high expression in early and late nauplii and early copepodites (Figure 11). Expression patterns in Na_V_1-II and Na_V_1-V were similar, with a peak in expression in the early copepodite, supporting the hypothesis that these two contigs represent partial sequences of a single gene. Since mapping of reads was limited to a single contig for each comp, it underestimated the total number of reads mappable to a given Na_V_1 gene. This might be particularly true for Na_V_1-I given the large number of splice variants predicted in this comp. Relative expression of the Na_V_ transcripts in the stages with the highest expression ranged from 3 to 17 RPKM. These expression levels are low in comparison to many of the lipid biosynthesis transcripts. The inclusion of RNA from the developmental stages in this transcriptome study may have been critical for predicting the Na_V_ proteins in *C. finmarchicus*. If the samples had been limited to adults and late copepodites, we might not have obtained enough coverage to assemble the transcripts.

Using *Calanus* sequences as blast probes to search the *Daphnia pulex* genome identified four genes with good E-values (<10^−82^). Only one (EFX89321) was annotated as a voltage-gated sodium channel and turned out to be a Na_V_2. A second, unidentified gene, EFX81393 referred to as Dappudraft 50150, matched both Calfi Na_V_1-I and *D. melanogaster* SCNA_DROME sequences with E = 0.0. Reciprocal blast of the remaining two returned calcium channels as top hits.

Thus, the current state of knowledge suggests that *Calanus* probably possesses three and possibly four distinct genes within the Na_V_1 family, each occurring in multiple isoforms, as contrasted to *Daphnia* and insects with a single such gene. The multiple sequences associated with the different comps is similar to the types of isoforms identified in *D. melanogaster*, and these alternatively spliced variants, if confirmed, may contribute to functional diversity as in the fruit fly. However, a cautionary note also emerged from this analysis: the alignment programs are designed to optimize the number of matches, but they may do this by splitting sequences. While permitting sequence splits as part of the alignment process is biologically valid (*e.g.* in alternative splice variants) [14], it may also lead to assembly artifacts. Thus, the alignments and segmentations deduced from this approach must be taken with some reservation, and need to be confirmed by other methods.

## Summary and Conclusions

Illumina sequencing of six multiplexed samples comprising different stages of *C. finmarchicus* provided a transcriptome for this non-model species and new insights into stage-specific expression patterns. The *de novo* transcriptome developed significantly expands genomic resources for this important species, which heretofore have been limited to fewer than 12,000 EST sequences [10]. This *de novo* transcriptome provides an opportunity for additional interpretation of previous studies based on qPCR, subtractive hybridization and microarray technologies [9]–[11], [52]–[54]. It adds to these studies by providing additional sequence data on the target transcripts, including predictions for full-length proteins, potential information on alternative splicing and even the presence of additional genes with similar function. Conversely, the EST database has been an invaluable tool for vetting the Trinity assembly, since these sequences are single pass reads on a Sanger sequencer.

Two transcriptomes were obtained from the *de novo* assembly. The complete assembly, composed of 206,041 contigs, includes many comps with multiple sequences. Using the voltage-gated sodium channel as an example, we determined that the multiple sequences within a comp represented different forms of presumably single genes. This analysis led to the hypothesis that multiplicity of function in the Na_V_1 channel in *C. finmarchicus* is achieved through both alternative splicing and gene duplication. This diversity of transcripts for voltage-gated sodium channels helps explain why these proteins have been so difficult to identify via molecular cloning. The hypothesized presence of multiple genes in the Na_V_1 family is the first reported for any member of the protostomes. This complete assembly, which represents coverage of ca. 65% of the transcriptome or better, provides an excellent tool for gene and protein discovery.

The reference transcriptome was composed of a single representative sequence obtained from each unique comp, and included 96,090 sequences. The reference transcriptome was annotated using global annotation tools, and it provided a useful tool for gene expression studies. In contrast to the complete transcriptome, mapping statistics using this reference resulted in a very low percentage of reads that mapped more than once (<1%). Mapping of the individual stages against the reference indicated that as much as 35 to 48% of the transcripts were not expressed in any particular developmental stage. Targeted analysis of expression pattern focused on transcripts involved in lipid biosynthesis suggests that transcripts with similar annotations may exhibit very different expression patterns during development. This is not surprising given the importance of lipids in developmental processes. Furthermore, two transcripts were highly expressed in the late copepodite (CV) stage. These lipid-rich individuals collected in July were most likely in a pre-diapause stage, and these transcripts may be important in the regulation of diapause.

## Supporting Information

Figure S1 Distribution of GOSlim annotations for biological process (A), molecular function (B) and cellular component (C). Blast2GO generated annotations produced GOSlim terms for 10,344 compounds, which are summarized in graphical format showing the number of annotations in each category.
(DOC)

Table S1 Assembly statistics. Statistics of *de novo* assemblies generated from subsets of *Calanus finmarchicus* RNAseq data using 1.5% to 100% of available reads using Trinity software.
(DOCX)

Table S2 Mapping statistics for six developmental stages in *Calanus finmarchicus*. Reads were mapped against the reference transcriptome of 96,090 comps. 100 bp long paired-end reads were trimmed by 9 bp prior to mapping. Forward and reverse reads were mapped separately to obtain technical replication.
(DOCX)
