# Supplementary material for: De Novo Assembly of a Transcriptome for Calanus finmarchicus (Crustacea, Copepoda) – The Dominant Zooplankter of the North Atlantic Ocean
Source: PLoS One. 2014 Feb 19;9(2):e88589. doi: 10.1371/journal.pone.0088589 (PMC3929608; doi:10.1371/journal.pone.0088589)

**Figure S1.** Distribution of GOSlim annotations for biological process (A), molecular function (B) and cellular component (C). Blast2GO generated annotations against SwissProt database produced GOSlim terms for 10,344 comps, which are summarized in graphical format showing the number of annotations in each category. 15 functions were not included in molecular function (B) graph, because they contained fewer then 100 comps. These included: chromatin binding (0003682; 96 comps); metal ion binding (0046872; 96 comps); cation binding (0043169; 96 comps); ion binding (0043167; 96 comps); calcium ion binding (0005509; 95 comps); translation factor activity, nucleic acid binding (0008135; 89 comps); carbohydrate binding (0030246; 89 comps); phosphoprotein phosphatase activity (0004721; 71 comps); phosphoric ester hydrolase activity (0042578; 71 comps); phosphatase activity (0016791; 71 comps); nuclease activity (0004518; 67 comps); electron carrier activity (0009055; 44 comps); antioxidant activity (0016209; 36 comps); neurotransmitter transporter activity (0005326; 23 comps); and translation regulator activity (0045182; 11 comps).


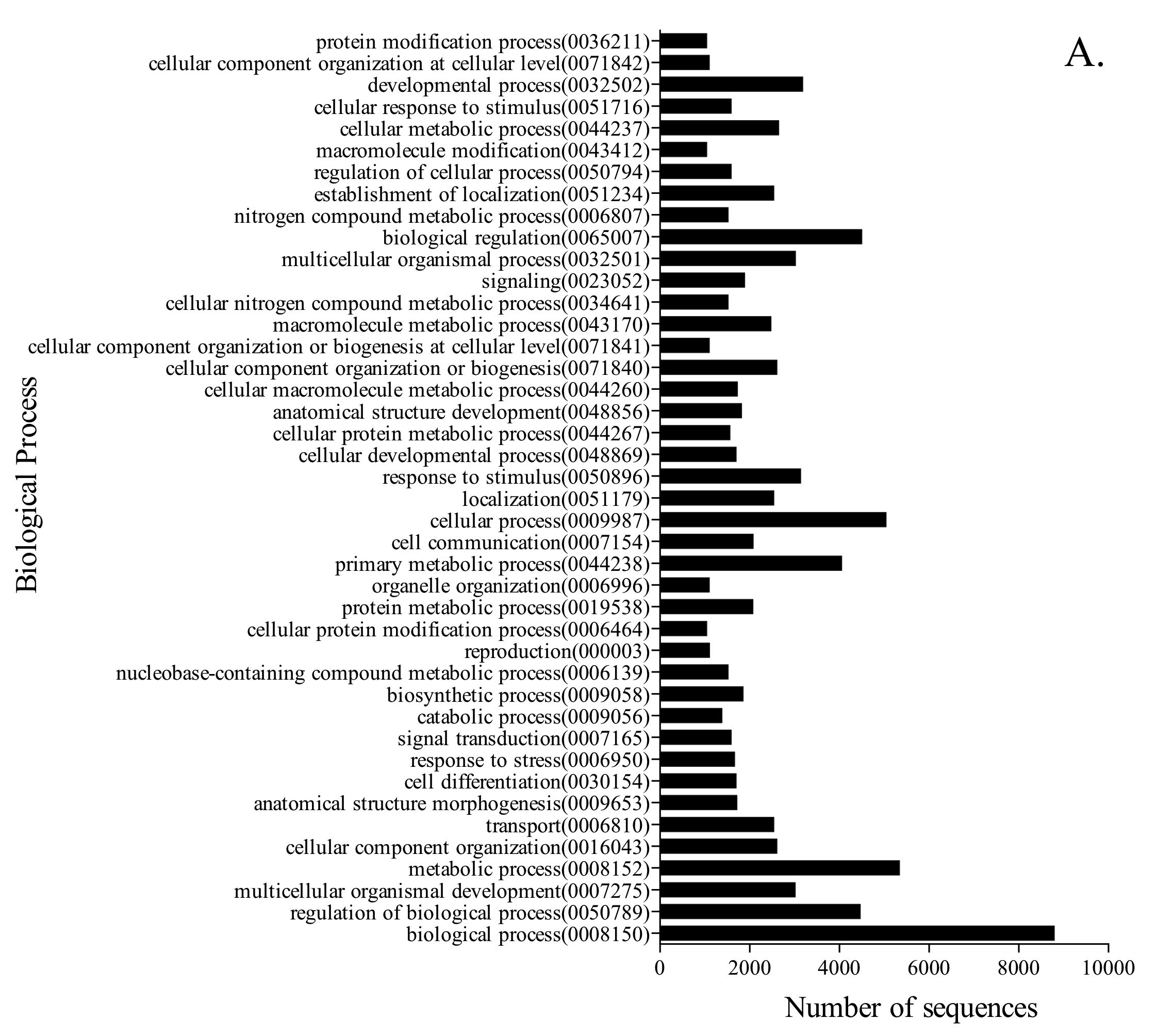


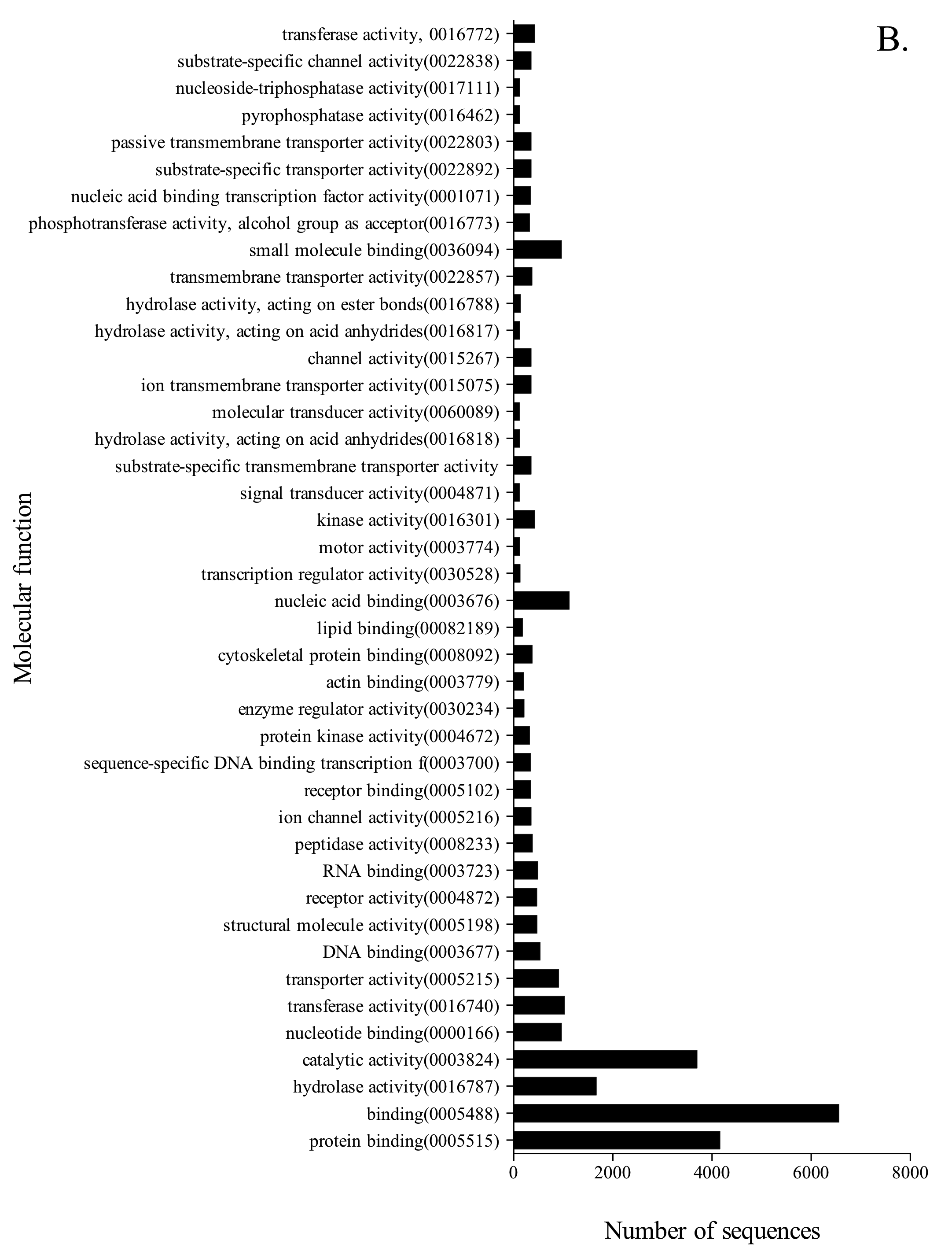


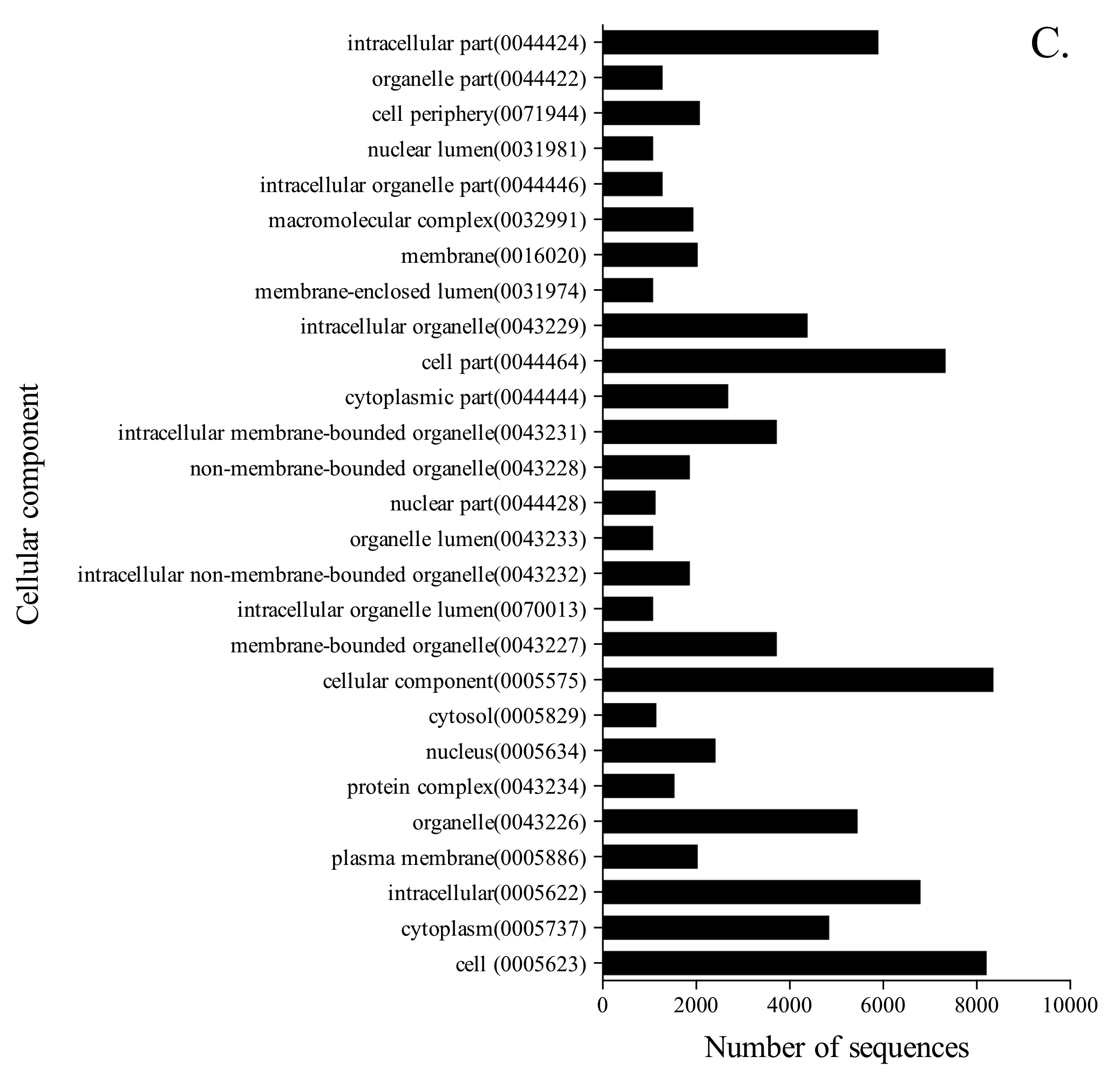

Supplement: Figure S1 — Distribution of GOSlim annotations for biological process (A), molecular function (B) and cellular component (C). Blast2GO generated annotations produced GOSlim terms for 10,344 compounds, which are summarized in graphical format showing the number of annotations in each category. (DOC) [file pone.0088589.s001.doc]
