# Supplementary material for: De Novo Assembly of a Transcriptome for Calanus finmarchicus (Crustacea, Copepoda) – The Dominant Zooplankter of the North Atlantic Ocean
Source: PLoS One. 2014 Feb 19;9(2):e88589. doi: 10.1371/journal.pone.0088589 (PMC3929608; doi:10.1371/journal.pone.0088589)
Supplement: Table S1 — Assembly statistics. Statistics of de novo assemblies generated from subsets of Calanus finmarchicus RNAseq data using 1.5% to 100% of available reads using Trinity software. (DOCX) [file pone.0088589.s002.docx]

**Table S1.** Statistics of *de novo* assemblies generated from subsets of *Calanus finmarchicus* RNAseq data using 1.5% to 100% of available reads using Trinity software.

|  | 1.5% of  reads | 3% of  reads | 6.25% of reads | 12.5% of reads | 25% of reads | 50% of reads | 75% of reads | 100% of reads |
| --- | --- | --- | --- | --- | --- | --- | --- | --- |
| Number of raw reads (million) | 6 | 12.5 | 25 | 50 | 100 | 200 | 300 | 400 |
| Assembled contigs (#) | 38,251 | 55,191 | 77,312 | 104,000 | 134,000 | 170,000 | 195,000 | 206,041 |
| Average length (bp) | 749 | 839 | 920 | 942 | 933 | 920 | 905 | 997 |
| Longest comp (bp) | 14,301 | 16,939 | 20,313 | 23,683 | 24,683 | 24,375 | 24,405 | 23,068 |
| Total length of assembly (bp) | 28,650,396 | 46,292,016 | 71,103,871 | 98,255,986 | 125,358,522 | 157,225,169 | 177,261,690 | 205,480,825 |
| Total GC count (bp) | 13,216,477 | 21,142,309 | 32,053,754 | 43,734,426 | 54,854,322 | 67,719,750 | 75,815,113 | 88,329,861 |
| GC Content for the whole assembly (%) | 46 | 46 | 45 | 44 | 44 | 43 | 43 | 43 |
| N50 length (bp) | 877 | 1,053 | 1,198 | 1,229 | 1,221 | 1,201 | 1,177 | 1,418 |
| N25 length (bp) | 1,624 | 1,923 | 2242 | 2,371 | 2,376 | 2,352 | 2,306 | 2,748 |
| N75 length (bp) | 509 | 581 | 646 | 663 | 573 | 639 | 626 | 701 |
| Unique comps (#) | 30,006 | 37,186 | 43,648 | 50,699 | 60,737 | 74,975 | 86,014 | 96,090 |
