# Supplementary material for: De Novo Assembly of a Transcriptome for Calanus finmarchicus (Crustacea, Copepoda) – The Dominant Zooplankter of the North Atlantic Ocean
Source: PLoS One. 2014 Feb 19;9(2):e88589. doi: 10.1371/journal.pone.0088589 (PMC3929608; doi:10.1371/journal.pone.0088589)
Supplement: Table S2 — Mapping statistics for six developmental stages in Calanus finmarchicus. Reads were mapped against the reference transcriptome of 96,090 comps. 100 bp long paired-end reads were trimmed by 9 bp prior to mapping. Forward and reverse reads were mapped separately to obtain technical replication. (DOCX) [file pone.0088589.s003.docx]

**Table S2**. Mapping statistics for six developmental stages in *Calanus finmarchicus*. Reads were mapped against the reference transcriptome of 96,090 comps. 100 bp long paired-end reads were trimmed by 9 bp prior to mapping. Forward and reverse reads were mapped separately to obtain technical replication.

| Library | Tech.  Rep. | Reads for mapping | Overall alignment rate | Total # reads mapped | Reads mapped 1 time | Reads mapped 1 time (%) | Reads mapped >1 time | Reads mapped >1 time (%) |
| --- | --- | --- | --- | --- | --- | --- | --- | --- |
| **Embryos** | 1 | 25,816,595 | 75.2 | 19,412,204 | 19,332,860 | 74.8 | 79,344 | 0.3 |
|  |  | 25,703,436 | 75.2 | 19,333,449 | 19,255,070 | 74.9 | 78,379 | 0.3 |
| **Early nauplius**  **(NI-NII)** | 1 | 31,573,258 | 75.6 | 23,882,395 | 23,631,137 | 74.8 | 251,258 | 0.8 |
|  | 2 | 31,254,273 | 75.5 | 23,607,898 | 23,362,141 | 74.7 | 245,757 | 0.8 |
| **Late nauplius**  **(NV-NVI)** | 1 | 31,662,241 | 77.1 | 24,403,803 | 24,141,364 | 76.2 | 262,439 | 0.8 |
|  | 2 | 31,702,164 | 77.0 | 24,418,351 | 24,159,701 | 76.2 | 258,650 | 0.8 |
| **Early copepodite**  **(CI-CII)** | 1 | 30,985,927 | 76.2 | 23,604,097 | 23,350,909 | 75.3 | 253,188 | 0.8 |
|  | 2 | 31,300,092 | 76.6 | 23,962,860 | 23,714,541 | 75.7 | 248,319 | 0.8 |
| **Late copepodite (CV)** | 1 | 33,675,136 | 73.4 | 24,711,471 | 24,487,318 | 72.7 | 224,153 | 0.7 |
|  | 2 | 33,216,645 | 73.4 | 24,395,429 | 24,174,696 | 72.7 | 220,733 | 0.7 |
| **Adult female** | 1 | 30,162,119 | 74.4 | 22,456,732 | 22,290,276 | 73.9 | 166,456 | 0.6 |
|  | 2 | 30,075,233 | 74.4 | 22,374,640 | 22,210,519 | 73.8 | 164,121 | 0.6 |
